# Cryoablation of primary breast cancer tumors induces a systemic abscopal effect altering TIME (Tumor Immune Microenvironment) in distant tumors

**DOI:** 10.3389/fimmu.2024.1498942

**Published:** 2024-12-02

**Authors:** Flávia Sardela de Miranda, Dalia Martinez-Marin, Rachel L. Babcock, Maribel Castro, Geetha P. Boligala, Sonia Y. Khan, Kathryn L. Furr, Isabel Castro-Piedras, Nicholas Wagner, Dakota E. Robison, Karla Daniele, Sharda P. Singh, Kevin Pruitt, Michael W. Melkus, Rakhshanda Layeequr Rahman

**Affiliations:** ^1^ Department of Surgery, School of Medicine, Texas Tech University Health Sciences Center, Lubbock, TX, United States; ^2^ Department of Immunology and Molecular Microbiology, School of Medicine, Texas Tech University Health Sciences Center, Lubbock, TX, United States; ^3^ Breast Center of Excellence, School of Medicine, Texas Tech University Health Sciences Center, Lubbock, TX, United States; ^4^ Department of Pharmacology, University of North Carolina at Chapel Hill, Chapel Hill, NC, United States; ^5^ Department of Cell Biology and Biochemistry, School of Medicine, Texas Tech University Health Sciences Center, Lubbock, TX, United States; ^6^ Department of Surgery, The University of Texas Rio Grande Valley (UTRGV) Rio Grande Valley, Harlingen, TX, United States; ^7^ Department of Pharmacology and Neuroscience, School of Medicine, Texas Tech University Health Sciences Center, Lubbock, TX, United States; ^8^ Department of Internal Medicine, School of Medicine, Texas Tech University Health Sciences Center, Lubbock, TX, United States; ^9^ Cancer Institute, MetroHealth System, Cleveland, OH, United States

**Keywords:** breast cancer, cryoablation, abscopal effect, RNA-seq analysis, immune response

## Abstract

**Introduction:**

Despite recent advances, triple-negative breast cancer (TNBC) patients remain at high risk for recurrence and metastasis, which creates the need for innovative therapeutic approaches to improve patient outcomes. Cryoablation is a promising, less invasive alternative to surgical resection, capable of inducing tumor necrosis via freeze/thaw cycles. Necrotic cell death results in increased inflammatory signals and release of preserved tumor antigens, which have the potential to boost the local and systemic anti-tumor immune response. Thus, compared to surgery, cryoablation enhances the activation of T cells leading to an improved abscopal effect, defined as the occurrence of a systemic response after local treatment. We previously showed with a bilateral-tumor mouse model of TNBC that cryoablation of the primary tumor leads to increased infiltration of distant (abscopal) tumors by tumor infiltrating lymphocytes (TILs) and decreased rates of recurrence and metastasis. However, the early drivers of the cryoablation generated abscopal effect are still unknown and knowledge of the mechanism could provide insight into improving the anti-tumor immune response through pharmacologic immune modulation in addition to cryoablation.

**Methods:**

One million 4T1-12B-luciferase expressing cells were transplanted into the mammary fat pad of BALB/c mice. Two weeks later, left (primary) tumors were either resected or cryoablated. A week after the procedure, right (abscopal) and left tumors, along with spleen, tumor-draining lymph node and blood were collected and processed for flow cytometry and/or RNA-sequencing and immunofluorescence.

**Results:**

Here we show that cryoablation of mouse mammary carcinomas results in smaller abscopal tumors that harbor increased frequencies of anti-tumor cells [such as natural killer (NK) cells], accompanied by a systemic increase in the frequency of migratory conventional type 1 dendritic cells (cDC1; CD103^+^ XCR1^+^), compared to resection. The changes in cell frequencies are mirrored by the immune gene signature of the abscopal tumors, with cryoablation inducing genes involved with NK cell activation and leukocyte-mediated toxicity, including IL11ra1 and Pfr1.

**Conclusions:**

These results better define the early mechanisms through which cryoablation improves tumor elimination, which is mediated by enhanced frequencies of anti-tumoral cells such as NK and cDC1s at the abscopal tumor and in the spleen of mice treated with cryoablation, respectively.

## 1 Introduction

The progression of cancer and development of metastasis is closely associated with the interrelation between cancer cells and their microenvironment, including immune responses – termed the tumor immune microenvironment (TIME). Innate and adaptive immunity are both involved with immune surveillance to eliminate pre-cancerous and cancerous cells, yet, transformed cells can evade immune control and generate immunosuppressive signals capable of modulating the activity of the immune system towards a pro-tumoral phenotype (1). Lymphocytes - including CD8^+^ cytotoxic and CD4^+^ helper T cells, natural killer (NK) and NKT cells – together with dendritic cells (DC) and pro-inflammatory macrophages (M1-like) have the power to induce an anti-tumoral response leading to tumor cell death. However, as tumor growth progresses, pro-tumoral populations of cells are recruited and generated at the tumor site, such as anti-inflammatory macrophages (M2-like), myeloid-derived suppressor cells [MDSCs, comprised of immunosuppressive monocytes, macrophages, and granulocytes (2)] and regulatory T (Treg) cells, all which act to counteract the anti-tumor response by suppressing the activity of effector cells and hampering the activation of newly recruited lymphocytes. Tumor cells also evade immunity by downregulating or losing expression of tumor antigens, releasing immunosuppressive molecules, and shedding soluble major histocompatibility complex (MHC)-I, among other mechanisms (1).

Collectively, the density and the diversity of the tumor-infiltrating cells affects prognosis and treatment response. For example, the levels of pro-inflammatory cytokines produced, and the degree of T cell infiltration are used to categorize tumors as either “cold” or “hot” (3). “Cold” tumors are non-T cell inflamed, characterized by T cell absence or exclusion, while “hot” tumors are T cell inflamed, characterized by T cell infiltration and molecular signatures of immune activation. This means “hot” tumors have higher response rates to immunotherapy (4, 5). Triple-negative breast cancer (TNBC) is, among all breast cancer subtypes, the most immunogenic (6–8), presenting with higher T cell infiltration, mutational burden, and PD-L1 expression (9, 10); however, when compared to other tumor subtypes, TNBC is considered a “cold” tumor (11–13). Despite recent advances in therapies, patients with TNBC remain at high risk for disease recurrence and early metastasis (14–17), with a combined 5-year relative survival rate of 77%; for patients with metastasized disease, that value lowers down to 12% (18). Therefore, innovative therapeutic strategies that leverage the immunogenicity of TNBC and enhance molecular signatures of immune activation provide an opportunity to improve patient outcomes.

**Figure 1:**
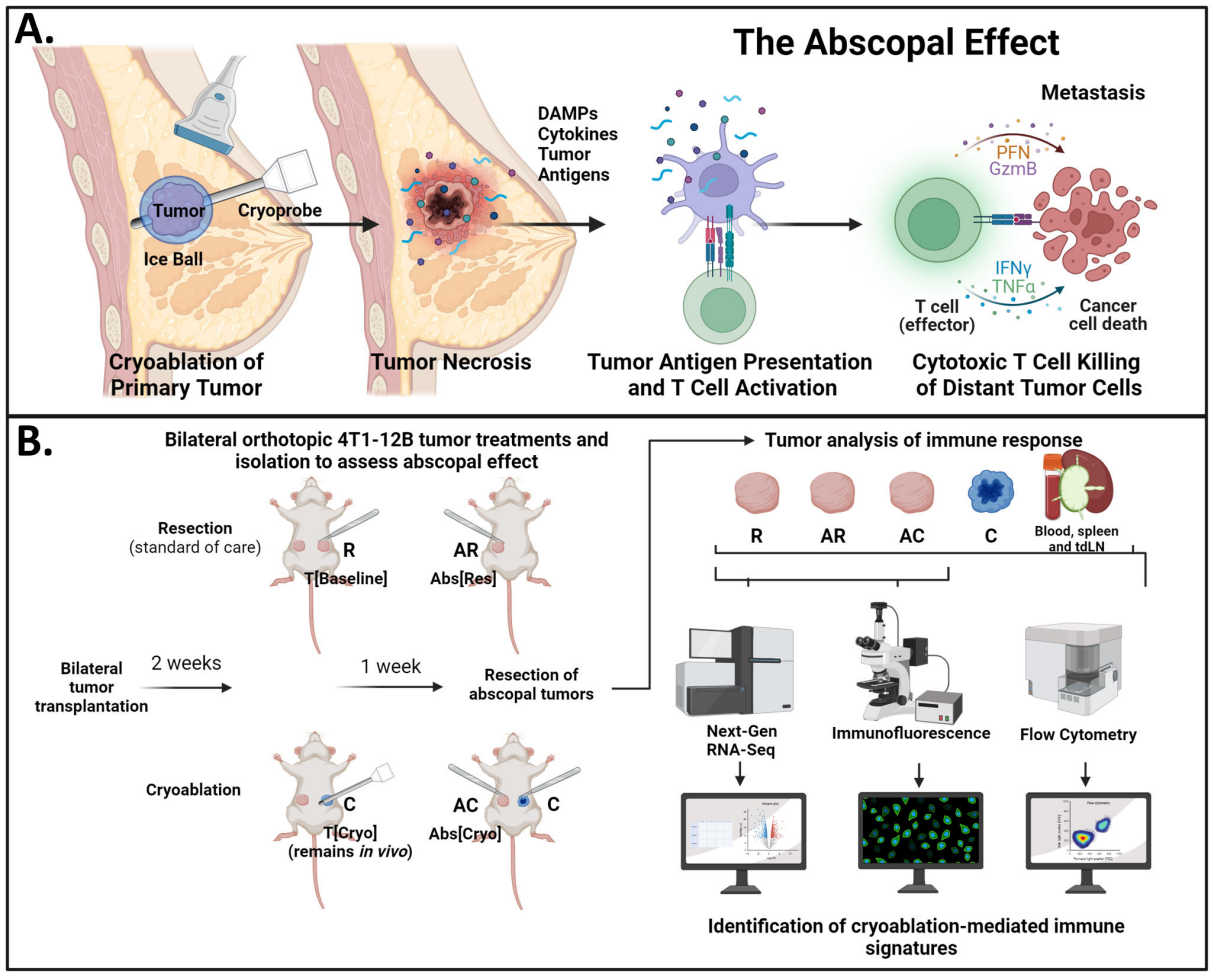
Experimental approach. **(A)** Schematic showing the breast cancer cryoablation procedure and the abscopal effect. Through a small incision on the skin, the cryoprobe is inserted into the tumor; as liquid nitrogen is released, an ice ball forms around the tumor, causing it to freeze. The freeze/thaw cycles lead to tumor necrosis, which results in release of preserved tumor antigens, damage associated molecular patterns (DAMPs), and inflammatory cytokines, which will be recognized by antigen presenting cells. The higher availability of inflammatory signals and tumor antigens will boost the activation of anti-tumor T cells that can recognize both local and distant tumor cells. The generation of a systemic response after a local treatment is called “abscopal effect”. **(B)** Schematic of experimental approach using our murine model of breast cancer. 1x10^6^ 4T1-12B cells were bilaterally transplanted in the mammary fat pad of BALB/c mice. Two weeks later, mice were divided into two groups and received their according treatment: resection or cryoablation. One-week post treatment, animals were sacrificed and cryoablated tumors, abscopal tumors, TdLNs, spleens, and peripheral blood were collected and processed for analysis. Created in BioRender. Melkus, M. (2024) BioRender.com/w50o762.

One promising approach is cryoablation, a method that kills tumor cells via rapid freeze/thaw cycles while leaving the dead tumor cells *in vivo*. The exposure of the tumor to subzero temperatures (≤ -40°C) results in tumor necrosis, an inflammatory type of cell death that promotes immune recruitment while preserving tumor-associated antigens (TAAs) (19–21). Since the ablated tumor (and consequently, its TAAs) remains *in vivo*, this introduces the possibility of augmenting the anti-tumor immune response, leading to an improved systemic immune activity, also known as the abscopal effect (**Figure 1A**). This contrasts with surgical resection, in which tumors and their TAAs are removed (21). Currently, cryoablation is clinically approved for the treatment of breast fibroadenomas and has shown promising results in low-risk breast cancer (22–26). There is great interest in applying this technique to treat TNBC, which is more immunogenic than the above-mentioned subtypes and, therefore, could benefit from the augmentation of the availability of TAAs and inflammatory signals.

Clinical studies have shown the long-term abscopal effects of cryoablation in hormone receptor positive breast cancer patients with lymph node and/or liver metastasis, which regressed by 5-6 months after the treatment (27, 28), however clinical data for TNBC remains limited. In a recent long-term pre-clinical pilot study from our group, we showed local treatment of TNBC by cryoablation increased systemic immune responses compared to surgical resection where, six weeks after the procedures, 40% of the mice in the resected group showed local recurrence and lung metastasis, while no recurrence or metastasis occurred in mice post-cryoablation (29). Other groups observed similar superior immune activation with cryoablation compared to resection in breast cancer (30) and colon cancer models (31). However, the early drivers of the cryoablation-generated abscopal effect in TNBC remain unknown. Knowledge of such information could help guide decisions regarding follow-up therapies for TNBC patients and support the development of combinational therapies for cryoablation, by supplying the field with clinically actionable data, such as key immune populations and gene pathways directly affected by cryoablation. This information could be used for identifying suitable immune modulators, such as ICIs, and choosing which is the most appropriate for combination with cryoablation, ultimately providing patients with more efficient and safer therapies that can enhance tumor elimination and immune surveillance, improving survival.

Herein, we used the bilateral-tumor murine breast cancer model, flow cytometry, immunofluorescence and RNA-sequencing (RNA-seq), to identify differences in immune cell frequencies and immune gene signatures after cryoablation of primary tumors. We observed global T cell immune responses in distant tumors post cryoablation, which may help guide follow-up and combinational therapy decisions. Furthermore, we were able to use RNA-seq deconvolution from paraffinized tissue to determine the frequency of immune populations in the primary resected tumor, which may aid in determining optimal biomarkers to predict patients’ response to cryoablation in a clinical setting.

## 2 Materials and methods

### 2.1 Cell lines

The luciferase-expressing triple-negative mammary carcinoma cell line, 4T1-12B, was obtained from TUFTS University (generously provided by Sahagian et al.) (32). Cells were cultured in Dulbecco’s Modified Eagle’s medium (DMEM) supplemented with 10% FBS and 1% penicillin/streptomycin, at 5% CO_2_.

### 2.2 Animals

Naïve female BALB/c mice, aged 8 – 11 weeks, were purchased from The Jackson Laboratory (RRID: IMSR_JAX:000651, Bar Harbor, NE, USA) and maintained in a pathogen-free environment within the animal laboratory animal care center (LARC) under a 12 hours light/12 hours dark cycle. Water and food (standard mouse chow) were provided ad libitum. Animals were regularly checked for health status. All experiments were conducted in accordance with the Institutional Animal Care and Use Committee (IACUC) at Texas Tech University Health Sciences Center policies and approved protocols.

### 2.3 Cryoablations and resections

The experimental approach is shown on **Figure 1B**. Naïve female BALB/c mice underwent bilateral orthotopic transplant with 1x10^6^ 4T1-12B cells per side. The left-sided tumor served as a primary tumor, used for treatment, while the right-sided tumor served as proxy to evaluate the potential abscopal effect, being referred in this paper as “abscopal tumor”.

**Figure 2:**
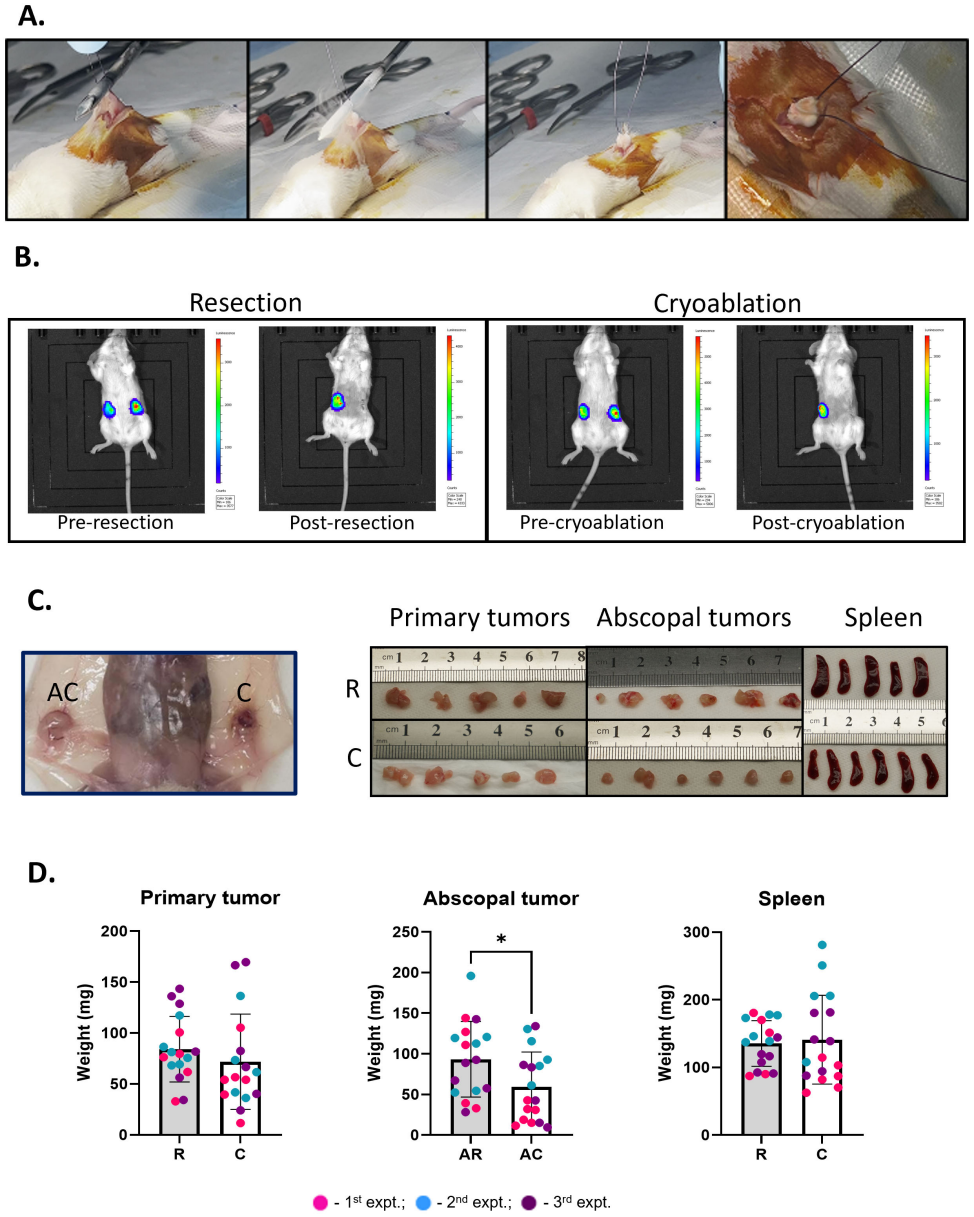
Abscopal tumors from cryoablation present lower weight compared to those from resection. **(A)** Cryoablation of the tumors. Skin is retracted away from the tumors, and the probe is laid flat on top of it. Far right panel shows a completely frozen tumor. **(B)** Representative IVIS pictures of mice from the resection and cryoablation groups, imaged 24 hours pre- and post-procedures. **(C)** Pictures of organs post-sacrifice. Far left panel shows the abscopal versus the cryoablated tumor in the mouse. The right panels show pictures of the primary tumors, abscopal tumors, and spleen, from left to right; top pictures are from the resection group, while bottom pictures are from the cryoablation group. **(D)** Graphs showing individual mouse tumor and spleen weights. The different dot colors indicate the experiment. Unpaired Student’s t-test for normally distributed data or Mann-Whitney test for non-normally distributed data was performed to compare resected vs cryoablation, with p<0.05 (*) considered significant. There is a one-week difference when tumors were isolated when comparing the resected to the cryoablated tumors (primary tumors). R, resection group; C, cryoablation group; AR, abscopal tumors from resection; AC, abscopal tumors from cryoablation. n = 17.

At two weeks, the mice were divided into 2 groups to where the average size of the left and right tumors were similar between groups. The left (primary) tumors were treated by either resection (standard of care group) or cryoablation (intervention group). Procedures were performed under strict aseptic technique, as we previously described (29). Surgical resection of the left tumors with grossly negative margins was performed on mice from the standard of care group, and resected tumors were used for downstream analysis (T[Baseline] or R). For the mice in the cryoablation group, the skin was incised and retracted away from the left tumor (T[Cryo] or C), and either the Visica 2 treatment system (Sanarus Technologies, Inc., Pleasanton, CA, USA) or the ProSense treatment system (IceCure Medical, Caesarea, Israel) probe connected to liquid nitrogen cryoablation machines were placed directly on top of the tumor mass; each tumor underwent two freeze/thaw cycles, and was left *in vivo* to induce the abscopal effect (**Figure 2A**). Freezing time was dependent on tumor size, while all tumors were equally given three minutes to thaw between freezing cycles. Inhaled 2.5% isoflurane anesthesia was used in both groups.

After completion of the surgical resection/cryoablation procedure, the skin was closed using 4-0 Prolene sutures (Ethicon, Somerville, NJ). All mice were administered 0.05 mg/kg of buprenorphine (Par Pharmaceuticals, Chesnut Ridge, NY) intraperitoneally in 200 µL of saline after the resection/cryoablation procedure. Additionally, one MediGel Hazeulnut (Clear H_2_O^®^, Portland, ME) supplemented with 0.5 mg/mL of Rimadyl (Zoetis, Parsippany, NJ) cup was provided per cage for pain management. Animals were monitored for 5 days for post-procedure complications. One-week post-treatment, the distant right tumors (Abs[Res] or AR and Abs[Cryo] or AC) and cryoablated tumors were resected for anti-tumor immunity analysis as previously reported (29).

Four independent experiments were conducted in accordance with TTUHSC-IACUC policies and approved protocols. The samples from one experiment (n=5) were used for RNA-seq and Immunofluorescence analysis, while the samples from other three experiments (n=5-6) were used for immune characterization by flow cytometry.

### 2.4 Tumor monitoring through IVIS

Twenty-four hours pre- and post-procedure (surgical resection or cryoablation), mice tumors were imaged with the *In vivo* imaging system (IVIS) Lumina XR (Caliper Life Sciences, PerkinElmer™) to evaluate the effectiveness of tumor resection/ablation. Mice received 100 µL of D-luciferin, Potassium- Salt (15 mg/mL) (GoldBio, St. Louis, MO) intraperitoneally (IP) and were imaged after 5 minutes, under 2.5% isoflurane anesthesia.

### 2.5 Mouse necropsy and cell isolation

One-week post-surgical resection or cryoablation, all mice were euthanized using CO_2_ followed by exsanguination via cardiac puncture, according to AVMA Guidelines for the Euthanasia of Animals:2020 Edition. The peripheral blood obtained through the cardiac puncture was collected into 0.5 M EDTA-containing tubes, along with the spleen, tumor-draining lymph nodes (TdLN; from both the primary and abscopal tumors), and abscopal/cryoablated tumors. Spleen and TdLNs were pressed through a 70 µm nylon mesh filter. Tumors collected on the sacrifice day, and resected tumors from the previous week, were either formalin-fixed and paraffin-embedded to make slides for RNA-seq and immunofluorescence or processed to obtain single cells for flow cytometry by mincing, digestion with 300 U/mL Collagenase, 100 U/mL Hyaluronidase, and 150 µg/mL DNAse, and filtering through a 70 µm mesh. Red blood cells were eliminated from the blood, tumor and spleen single-cell suspensions using red blood cell lysis buffer (STEMCELL Technologies, Vancouver, Canada).

### 2.6 Flow cytometry analysis for immune phenotyping

Isolated mononuclear cells from blood, tumors, TdLNs and spleens had their Fc receptors blocked with CD16/32 antibody mix (Invitrogen, San Diego, CA) and were stained with antibodies for our established lymphoid and myeloid mouse panel (**Table 1**) and the Live/Dead Aqua viability stain (Thermo Fisher Scientific Inc., Waltham, MA). Flow data was acquired using a Cytek Northern Lights™-Clinical (NL-CLC) spectral analyzer (Cytek Biosciences, Freemont, CA) and the SpectroFlo^®^ software. Data was analyzed using FlowJo v10.8.1 software.

**Table 1:** Antibodies used for flow cytometry.

	Antibody	Clone	Flurophore	Catalog number	Manufacturer	Concentration
Lymphocyte panel	NKG2A	20d5	APC	564383	BD	1:100
	CD3	17A2	APC-Cy7	100222	Biolegend	1:50
	CD45	30-F11	AF700	103128	Biolegend	1:100
	B220	RA3-6B2	BV421	103240	Biolegend	1:50
	ICOS	C396.4A	BV605	567920	BD	1:100
	CD8	53-6.7	BV650	100742	Biolegend	1:100
	CD107a	1D4B	BV711	564348	BD	1:200
	CD127	SB/199	BV786	563748	BD	1:200
	CD11b	M1/70	FITC	11-0112-85	eBioscience	1:200
	CD25	PC61	PE	12-0251-83	eBioscience	1:100
	CD62L	MEL-14	PE-CF594	562404	BD	1:200
	CD4	GK1.5	PE-Cy7	25-0041-82	invitrogen	1:200
	CD44	IM7	PerCP-Cy5.5	560570	BD	1:50
	CCR3	J073E5	APC	144512	Biolegend	1:100
Myeloid Panel	CD8a	53-6.7	APC-Cy7	100714	Biolegend	1:100
	CD45	30-F11	AF700	103128	Biolegend	1:50
	XCR1	ZET	BV421	148216	Biolegend	1:100
	Ly6C	HK1.4	BV605	128036	Biolegend	1:200
	MHCII	M5/114.15.2	BV650	107641	Biolegend	1:100
	IL-5Ra	T21	BV711	740817	BD	1:200
	CD103	2.00E+07	BV785	121439	Biolegend	1:100
	CD11b	M1/70	FITC	11-0112-85	eBioscience	1:200
	CD172a	P84	PE	144012	Biolegend	1:200
	Siglec-F	E-50-2440	PE-CF594	562757	BD	1:200
	CD11c	N418	PE-Cy7	117318	Biolegend	1:100
	Ly6G	1A8	PerCP-Cy5.5	560602	BD	1:50

For analysis, debris and doublets were excluded, and live immune cells were selected by gating on the CD45^+^ and live-and-dead negative population. In the lymphocyte panel (**Supplementary Figure 1A**), B cells were defined as B220^+^, myeloid cells were CD11b^+^, NK cells were NKG2A^+^, and T cells were CD3^+^. We further gated the CD3^+^ population on NKG2A^+^ (NKT cells), CD4^+^ (helper T cells), and CD8^+^ (cytotoxic T cells). CD4^+^ and CD8^+^ T cells were considered activated based upon inducible T cell costimulator (ICOS) expression, and subpopulations were defined based on the expression of CD62L and CD44, where: CD62L^+^CD44^-^ are Naïve T cells (P1); CD62L^+^CD44^+^ are central memory T cells (P2); CD62L^-^CD44^+^ are effector/effector memory T cells (P3); and CD62L^-^CD44^-^ are pre-effector T cells (P4) (33). Regulatory T cells (Tregs) were identified as CD4^+^, CD127^low^ and CD25^high^, and CD107a expression was used to identify CD8^+^ T cells that secreted perforin. In the myeloid panel (**Supplementary Figure 1B**), conventional DCs (cDC) were defined as CD11c^+^ and MHCII^high^ and then assessed to separate XCR1^+^ type 1 cDCs (cDC1) and CD172α^+^ type 2 cDCs (cDC2). cDC1s were further classified into migratory or lymphoid-resident cells based on CD103 or CD8α expression, respectively. The CD11b^+^ Ly6G^+^ population was defined into 2 subpopulations: Ly6G^high^ (Siglec-F^low^) and Ly6G^intermediate^ (Siglec-F^high^) neutrophils (34–36), the latter being classically defined as immunosuppressive neutrophils in lung cancer and acute spleen infection studies (37–40). Finally, CD11b^+^, Ly6G^-^, Siglec-F^-^ monocytes and macrophages were defined based on Ly6C and MHCII expression: M2-like TAMs were defined as Ly6C^low^ and MHCII^low^; M1-like TAMs were Ly6C^low^ and MHCII^high^; inflammatory monocytes were Ly6C^high^ and MHCII^low^; and immature macrophages were Ly6C^+^ and MHCII^high^ (41, 42).

### 2.7 Bulk tumor RNA-sequencing and analysis pipeline

Tumor RNA was purified from formalin-fixed, paraffin-embedded tissue sections using the RNeasy FFPE Kit (Qiagen, Cat #73504). rRNA was depleted for mRNA enrichment and the QIAseq Stranded Total RNA Lib Kit (Qiagen, Cat #180745) was used to construct the strand-specific RNA-seq library. Whole transcriptome sequencing was performed on an Illumina NextSeq 500 instrument. Differential gene expression analysis was done using EdgeR and its associated packages, and quality control (QC) with FastQC and Cutadapt. FASTQ files were trimmed and mapped to GRCm39/mm39 using HISAT2. Mapped sequences (number of reads per annotated genes) were counted with feature Counts. For each step, QC reports were aggregated using MultiQC. Read counts were annotated using biomaRt (ENSEMBL); lowly expressed genes were filtered prior to further analysis. *Voom* was used as QC to maintain the false discovery rate below the nominal rate. To eliminate biases between libraries, normalization for composition bias was performed with EdgeR. Differential expression and gene set testing were performed with limma and visualized with Glimma. Ingenuity Pathway Analysis (IPA), REACTOME, GO, GSEA, and Cibersort were used for further analysis of activated signaling pathways and identification of gene targets. RNA-sequencing data is available in Gene Expression Omnibus (https://www.ncbi.nlm.nih.gov/geo/).

### 2.8 Statistical analysis

**Figure 3:**
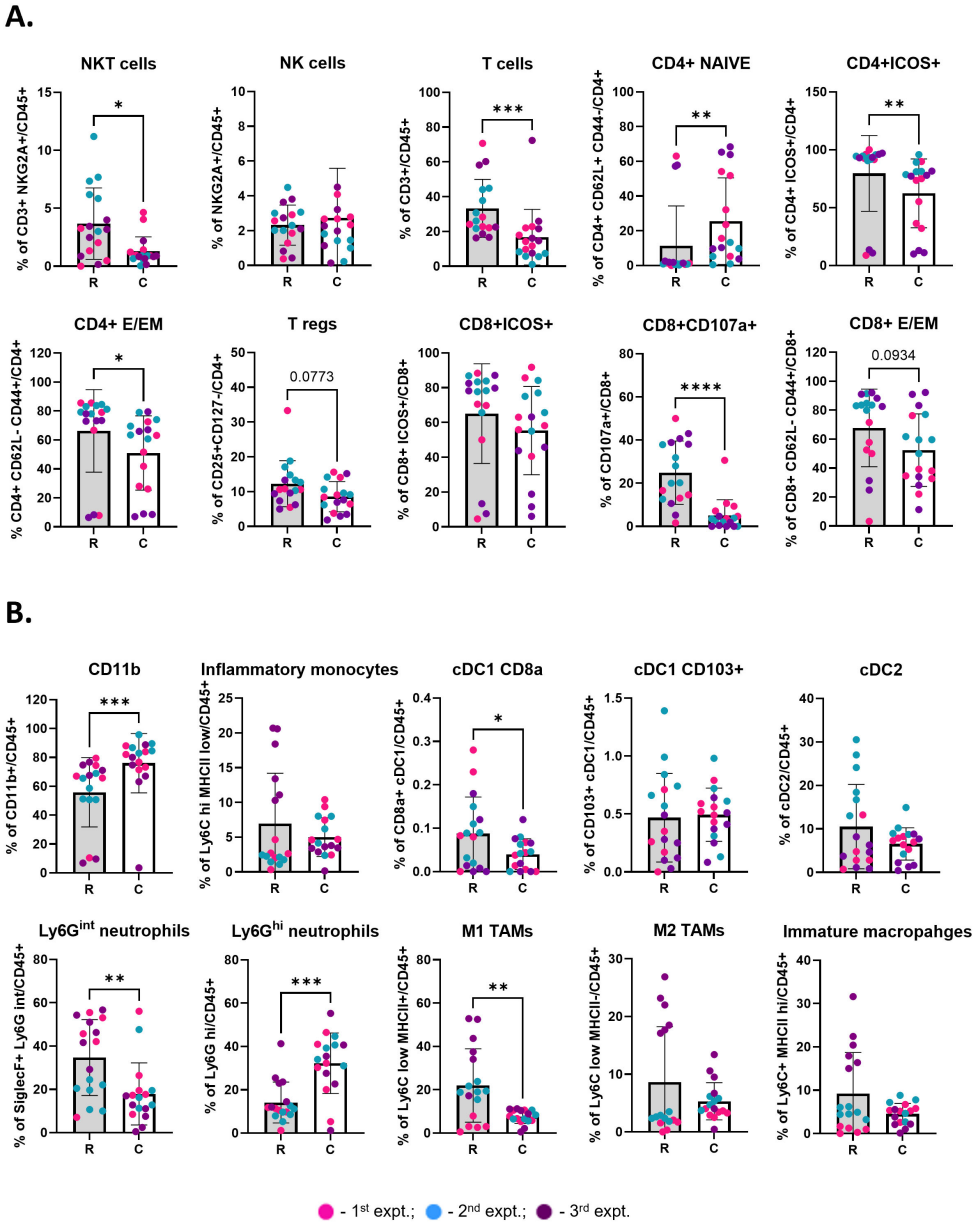
Cryoablation of primary tumor leads to enhanced local infiltration by naïve and inflammatory cells one-week after the procedure. **(A)** Lymphoid populations at the primary tumors. Subpopulations of T cells were analyzed as frequency of the parent (CD4^+^ or CD8^+^), while the parent immune populations were analyzed as frequency of the live immune cells (CD45^+^). **(B)** Myeloid populations at the primary tumors, analyzed as frequency of the live immune cells (CD45^+^). The different dot colors indicate the experiment. Unpaired Student’s t-test for normally distributed data or Mann-Whitney test for non-normally distributed data was performed comparing resected vs cryoablated tumors, with p<0.05 (*), p<0.01 (**), p<0.001 (***) and p<0.0001 (****) considered significant. There is a one-week difference when tumors were isolated when comparing the resected to the cryoablated tumors (primary tumors). R, resection group; C, cryoablation group; CM, central memory; E/EM, effector/effector memory. n = 17.

**Figure 4:**
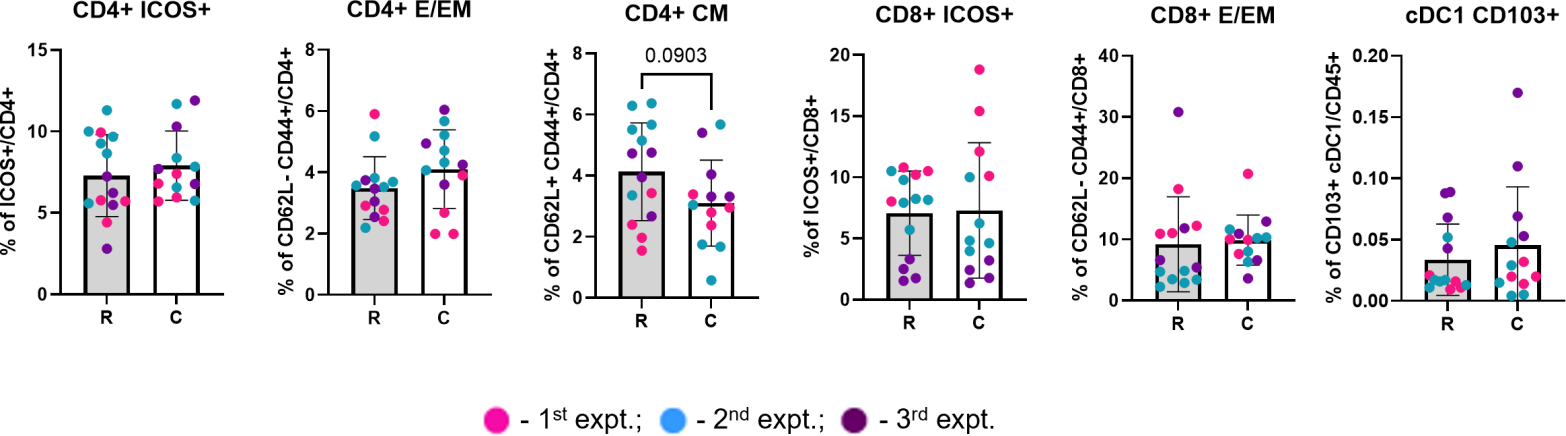
TdLNs from the cryoablated and resected tumors present similar frequency of immune populations. Graphs depict the frequency of different lymphocyte and DC populations at the lymph nodes draining treated tumors, analyzed as frequency of the parent (CD4^+^ or CD8^+^) and frequency of the live immune cells (CD45^+^), respectively. The different dot colors indicate the experiment. Unpaired Student’s t-test for normally distributed data or Mann-Whitney test for non-normally distributed data was performed comparing lymph nodes draining the resected vs cryoablated tumors. All lymph nodes were collected at 3 weeks. R, resection group; C, cryoablation group; CM, central memory; E/EM, effector/effector memory. n = 13.

**Figure 5:**
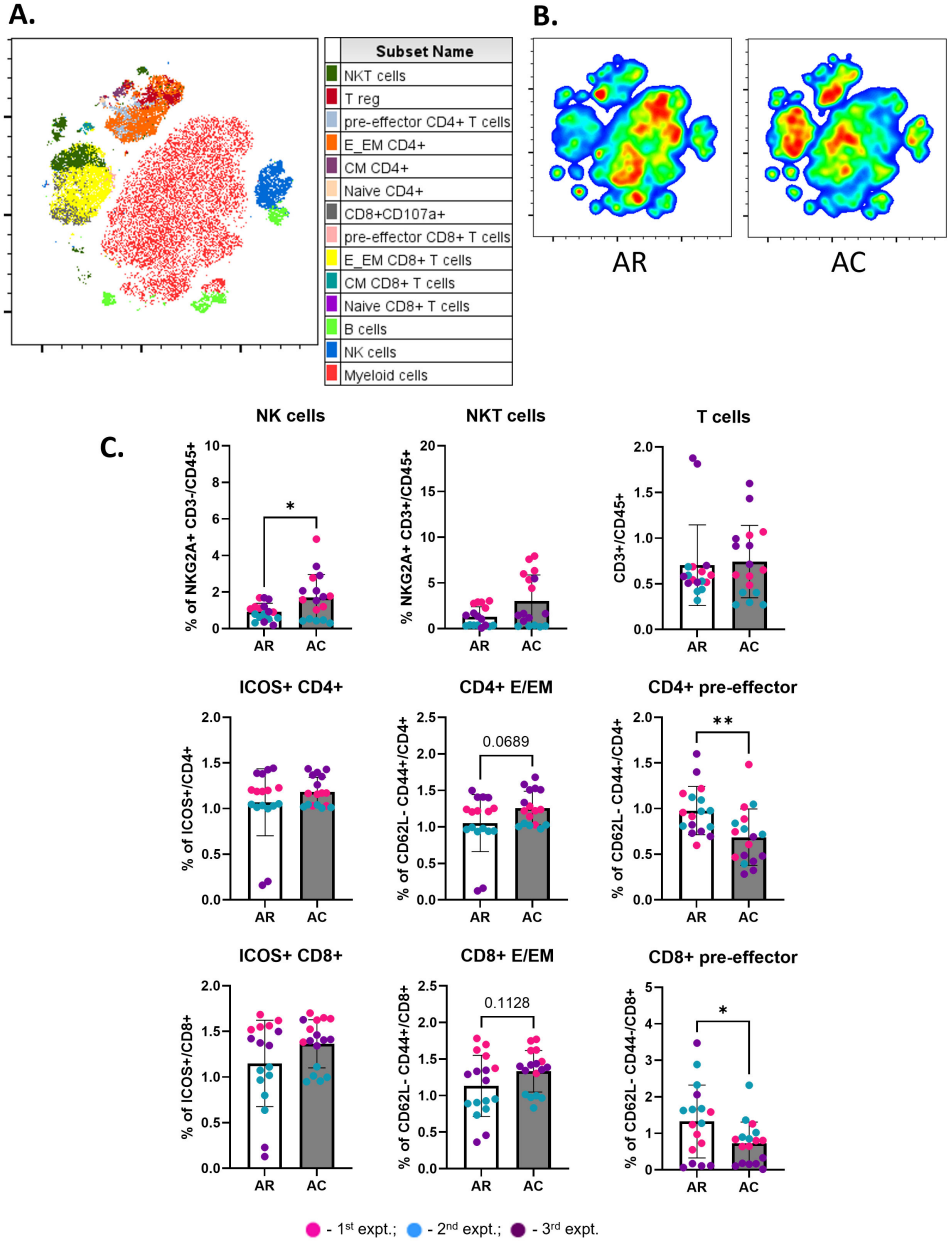
Abscopal tumors from cryoablation present increased frequencies of anti-tumor cells. **(A)** Representative tSNE map from one experimental repeat, showing the cluster localization of different lymphoid populations at the abscopal tumors. **(B)** Representative tSNE heatmap from one experimental repeat for concatenated abscopal tumors from resection (AR) and abscopal tumors from cryoablation (AC) samples. **(C)** Frequency of lymphoid populations on abscopal tumors from resection and cryoablation. Parent immune populations were analyzed as frequency of the live immune cells (CD45^+^), while subpopulations of T cells were analyzed as frequency of the parent (CD4^+^ or CD8^+^). The frequencies of each immune population were normalized by the average of the resected tumor frequencies, matched by experiment. The different dot colors indicate the experiment. Unpaired Student’s t-test for normally distributed data or Mann-Whitney test for non-normally distributed data was performed to compare resection vs cryoablation, with p<0.05 (*) and p<0.01 (**) considered significant. AR, abscopal from resection; AC, abscopal from cryoablation; CM, central memory; E/EM, effector/effector memory. n = 17.

**Figure 6:**
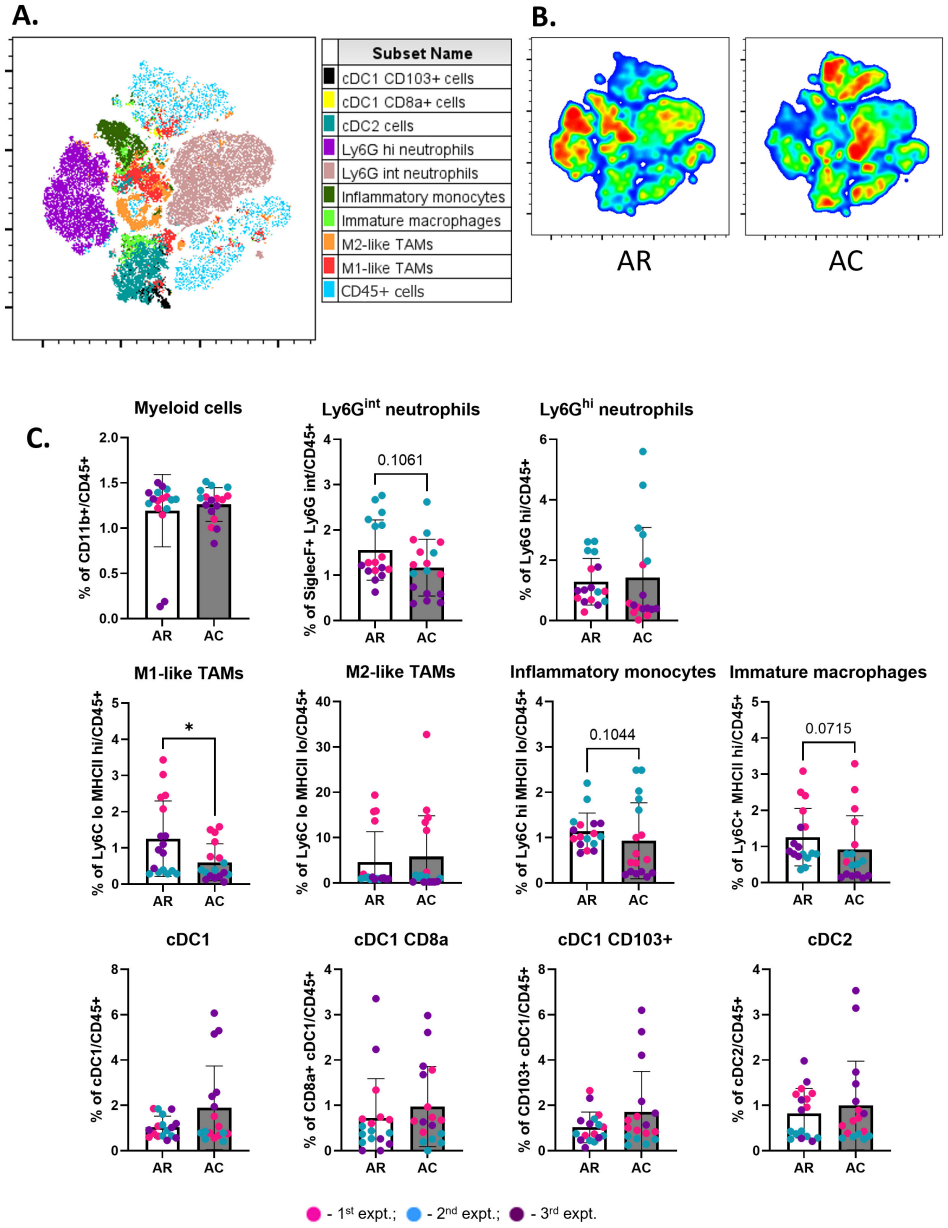
Cryoablation decreases immunosuppression in abscopal tumors. **(A)** Representative tSNE map from one experimental repeat, showing the cluster localization of different myeloid populations at the abscopal tumors. **(B)** Representative tSNE heatmap from one experimental repeat for concatenated abscopal tumors from resection (AR) and abscopal tumors from cryoablation (AC) samples. **(C)** Frequency of myeloid populations on abscopal tumors from resection and cryoablation. All immune populations were analyzed as frequency of the live immune cells (CD45^+^). The frequencies of each immune population were normalized by the average of the resected tumor frequencies, matched by experiment. The different dot colors indicate the experiment. Unpaired Student’s t-test for normally distributed data or Mann-Whitney test for non-normally distributed data was performed to compare resection vs cryoablation, with p<0.05 (*) considered significant. AR, abscopal from resection; AC, abscopal from cryoablation. n = 17.

**Figure 7:**
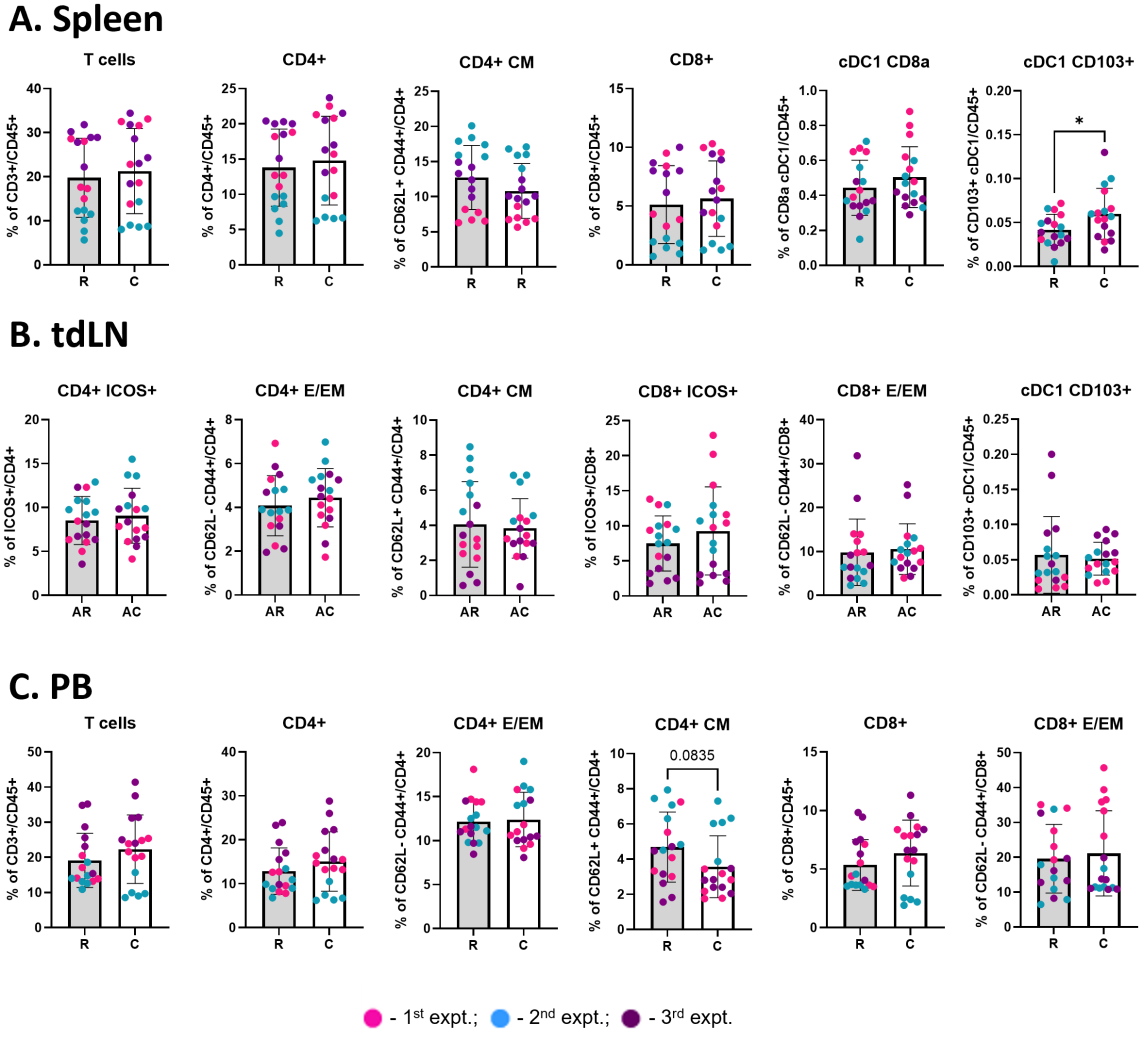
Cryoablation boosts the generation of migratory cDC1s. Graphs show the frequency of immune populations at the spleen [**(A)**, n = 17], abscopal tumor-draining lymph node [**(B)**, n = 17], and peripheral blood [**(C)**, n = 17]. All immune populations were analyzed as frequency of the live immune cells (CD45^+^), except for the CD4^+^ and CD8^+^ subpopulations (analyzed as frequency of the parent). The different dot colors indicate the experiment. Unpaired Student’s t-test for normally distributed data or Mann-Whitney test for non-normally distributed data was performed comparing resection vs cryoablation, with p<0.05 (*) considered significant. R, resection group; C, cryoablation group; AR, abscopal from resection; AC, abscopal from cryoablation; CM, central memory; E/EM, effector/effector memory.

Statistical analyses were performed using GraphPad Prism software version 9.00 for Windows (La Jolla CA., www.graphpad.com). Normally distributed data was analyzed by unpaired t-test or one-way ANOVA with Tukey’s post-test, when two or three unrelated groups were analyzed together, respectively. Mann-Whitney t-test and Kruskal Wallis with Dunn’s post-test were used to analyze non-normally distributed data. Differences were considered statistically significant when *p<0.05, ** *p*<0.01, ***p<0.001, and ****p<0.0001, with confidence interval (CI) of 95%. Numerical *p* values are shown in some circumstances to indicate trends. All quantitative flow cytometry analysis was performed with 5-6 animals per group, from three different experiments (in the graphs from **Figures 2**–**7**: 1^st^ experiment – pink dots; 2^nd^ experiment – blue dots; 3^rd^ experiment – purple dots). RNA-seq and immunofluorescence analysis were performed with n=5 animals per group, from one experiment.

## 3 Results

### 3.1 Cryoablation of primary tumor completely kills the tumor mass and leads to reduced abscopal tumor weight

To evaluate early immune changes due to cryoablation, luciferase expressing 4T1 tumor cells were bilaterally transplanted into the mammary fat pad of BALB/c mice. Two weeks after, all mice had successfully developed bilateral tumors and were treated with resection (standard of care) or cryoablation. For cryoablation, freezing time was tumor size dependent, with liquid nitrogen being continuously released until tumors were completely frozen (**Figure 2A**); the cryoablated tumors remained *in vivo* until the end of the experiment. Mice were administered luciferin and underwent IVIS imaging system 24 hours pre- and post-procedures (**Figure 2B**). In all mice, tumors were vividly visible with luciferase activity prior to therapy; all activity disappeared with both resection and cryoablation of the primary tumors post-treatment (**Figure 2B**).

To identify early events in the anti-tumor immune response, mice were sacrificed at 1-week post primary treatment and tissues collected for analysis (**Figure 2C**). Assessment of tumor weights revealed abscopal tumors from cryoablation were significantly smaller than abscopal tumors from resection (AC = 59.26 ± 42.82 and AR = 93.32 ± 46.59, respectively), while the weights of primary tumors (R = 84.09 ± 32.22; C = 71.74 ± 46.80) and spleen (R = 135.4 ± 33.74; C = 140.9 ± 65.67) showed no significant differences between the groups (**Figures 2C, D**). These observations suggest that cryoablation of primary tumor results in systemic effects, influencing distant tumor’ size.

### 3.2 Cryoablation induces an early inflammatory response characterized by naïve T cell infiltration in primary tumors

Cryoablation kills both tumor and non-tumor cells (including immune cells). Thus, we were interested in analyzing the pattern of infiltration by responding immune cells at the cryoablated tumors one-week after treatment, to understand the initiation of the abscopal effect. Through analysis of the immune landscape of primary resected tumors, collected at two weeks (R), and cryoablated tumors, collected at three weeks (C), by flow cytometry, we found a shift in cryoablated tumors, with increased recruitment of CD4^+^ naïve T cells (**Figure 3A**) and Ly6G^hi^ neutrophils (**Figure 3B**) one-week post-cryoablation. Neutrophils are especially important for cleaning and repairing the ablated site, while the newly recruited CD4^+^ naïve T cells can be activated by antigen presenting cells and aid in the elimination of tumor cells. Additionally, we observed a significant decrease in the frequency of multiple lymphoid populations in the cryoablated tumors, such as infiltrating NKT cells, T cells, other subpopulations of CD4^+^ T cells (Naïve, ICOS^+^, effector/effector memory), and subpopulations of CD8^+^ T cells (CD107a^+^ and effector/effector memory) (**Figure 3A**), as well as myeloid populations, such as CD8a^+^ XCR1^+^ (resident) DCs, Ly6G^int^ neutrophils, and M1-like macrophages (**Figure 3B**) compared to the resected baseline control tumor. The decreased frequency of these populations reflects the significant increase in the frequency of CD11b^+^ myeloid cells at the cryoablated tumor (**Figure 3B**). More importantly, the percentage of these immune cells in the cryoablated tumor is clinically significant since they represent new tumor infiltrating immune cells.

DCs capture antigens in the tumor and transport them to the TdLN to activate T cells. At the lymph node draining the primary (treated) tumor, collected one-week after resection/cryoablation, we observed a trend for having more migratory cDC1s (CD103^+^ XCR1^+^) in the cryoablation group, compared to resection, while most other populations seemed to present similar frequencies between the 2 groups (**Figure 4**). Not surprisingly, we also observed a trend for having lesser central memory CD4^+^ T cells, which was expected at this early time-point (**Figure 4**).

Altogether, these results demonstrate the ability of cryoablation to induce an inflammatory response that has the potential to generate an abscopal effect, by recruiting more naïve cells and boosting antigen presentation.

### 3.3 Cryoablation of primary tumors shifts the TIME at abscopal tumors towards an enhanced anti-tumoral state

For analysis of the immune populations at abscopal tumors (collected at three weeks), the frequencies of each experiment were normalized to the average frequencies of the control resected tumors (collected at two weeks) from the same experiment, to minimize the variance between experiments due to exterior factors. Raw immune frequencies from abscopal tumors, not normalized to the resected tumors, are available in **Supplementary Figure 2**.

Live immune cells from abscopal tumors were analyzed by t-distributed stochastic neighbor embedding (t-SNE), an unsupervised non-linear dimensionality reduction technique to explore and visualize the data. Lymphoid populations successfully clustered together based on similarity (**Figure 5A**, **Supplementary Figure 3**). Abscopal tumors from resection (AR) showed greater enrichment of myeloid cells, whereas abscopal tumors from cryoablation (AC) showed enrichment of T cells (CD4^+^ and CD8^+^) and NK cells (**Figure 5B**). Quantitative analysis showed a significant increase in the frequency of NK cells in the abscopal tumors from cryoablation compared to abscopal tumors from resection, and similar trends for other important populations, such as CD4^+^ and CD8^+^ effector/effector memory cells (**Figure 5C**). Interestingly, there was a significant decrease in the frequency of CD4^+^ and CD8^+^ pre-effector cells, suggesting that cryoablation of primary tumor may be accelerating the development of effector/effector memory T cells in abscopal tumors compared to resection (**Figure 5C**). The frequencies of NKT and T cells, and ICOS^+^ CD4^+^ and CD8^+^ T cells were similar between both groups (**Figure 5C**).

Myeloid populations also successfully clustered together (**Figure 6A**, **Supplementary Figure 3**). Different myeloid populations were enriched between abscopal tumors from resection and cryoablation, with inflammatory monocytes and M1-like TAMs being enriched in abscopal tumors from resection (**Figure 6B**). Quantitative analysis showed that abscopal tumors from cryoablation tended to have reduced inflammatory cell infiltration, characterized by a significant decrease in the frequency of M1-like TAMs, and overall trends for reduced Ly6G^int^ neutrophils, inflammatory monocytes (Ly6C^hi^ MHC-II^lo^), and immature macrophages (Ly6C^+^ MHC-II^hi^) (**Figure 6C**). We also observed a trend for increased frequencies of multiple DC populations (**Figure 6C**).

These results indicate that cryoablation is positively influencing the abscopal tumor, by leading to increased frequencies of anti-tumor cells, and decreased frequencies of pro-tumoral cells when compared to abscopal tumors from resection.

### 3.4 Cryoablation enhances cDC1 infiltration in the spleen

We observed a significant increase in the frequency of migratory (CD103^+^ XCR1^+^) cDC1 cells in the spleens of mice from the cryoablation group, compared to resection (**Figure 7A**), which suggests cryoablation may result in activation of T cells at the periphery by enhancing DC activity. Considering that the animals were sacrificed one-week post-resection/cryoablation to allow analysis of early immune responses, it is promising to observe increase in the frequency of a population so important for immune activation in response to the cryoablation treatment. Frequencies of other immune populations analyzed at the spleen, lymph nodes draining abscopal tumors, and blood remained unchanged (**Figures 7A–C**).

### 3.5 Cryoablation of primary tumor modifies the expression of immune-related genes at abscopal tumors, altering important anti-tumor pathways

**Figure 8:**
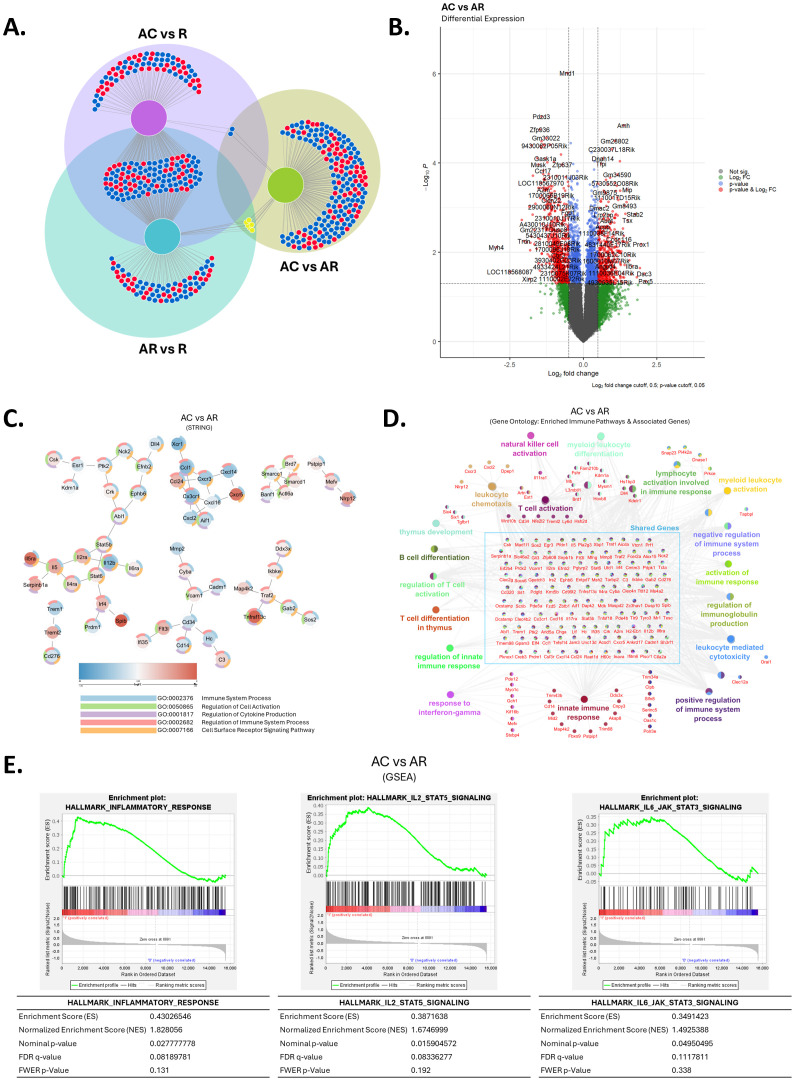
Cryoablation of primary tumor modifies the immune signature of distant tumors. **(A)** Venn diagram for differentially expressed genes for each tumor treatment condition compared to baseline control tumors. **(B)** Differential gene expression analysis on RNA sequencing of abscopal tumors from cryoablation in comparison to abscopal tumors from resection. **(C)** STRING analysis of abscopal tumors from cryoablation versus abscopal tumors from resection. **(D)** Gene Ontology of enriched immune pathways and associated genes in abscopal tumors from cryoablation, compared to abscopal tumors from resection. **(E)** Gene set enrichment analysis (GSEA) of significantly enriched immune pathways in abscopal tumors from cryoablation, compared to abscopal tumors from resection. R, resected tumor; AR, abscopal tumor from resection; AC, abscopal tumor from cryoablation. n = 5.

To investigate the gene expression differences underlying the previously described results and to provide knowledge to guide follow-up therapy choices in the clinical settings, we conducted Next-Generation RNA-Sequencing with resected and abscopal tumors. Globally, cryoablation induces both similar and unique changes in the abscopal tumor gene signature compared to resection (**Figure 8A**). Abscopal tumors from cryoablation displayed numerous significantly upregulated (1136) and downregulated (1406) genes compared to those from resection (**Supplementary Table 2**), demonstrating the systemic effects of cryoablation at the gene level (**Figure 8B**).

We used pathways analyses to define the immune-related changes between abscopal tumors from cryoablation and resection. STRING analysis revealed regulation of important genes involved with cell activation, cytokine production and receptor signaling pathways, with some differentially expressed genes (DEGs) suggesting cryoablation positively affected the anti-tumor activity, such as downregulation in the expression of *Cd276*, *Cd34*, *Mmp2*, and the pro-tumoral cytokine *Ccl1*, and up-regulation of *Il5ra* and *Nlrp12*. Other DEGs suggest negative modulation of anti-tumor activity, including enrichment of *Cxcr5* and *Cadm1*, genes linked with tumor development, progression, or migration, and down-regulation of *Xcr1*, a gene specific for the cDC1 subset (**Figure 8C**). Notably, we observed infiltration of anti-tumor immune cells in the abscopal tumors from cryoablation compared to resection (**Figure 5**), indicating that modulation of these genes does not adversely affect immune frequencies at the tumor sites.

Gene Ontology analysis further revealed upregulation of genes involved with NK cell activation, lymphocyte activation, leukocyte chemotaxis, leukocyte mediated toxicity, and innate immune response, among others (**Figure 8D**). Some upregulated genes worth highlighting are *Pfr1* (encodes perforin), and *Il2ra*, which are involved with CD8^+^ T cell cytotoxicity and T cell activation, respectively (**Figure 8D**). We tested the protein expression of some of these genes and others associated with T and NK cell cytotoxicity (CD4, CD8, IFN-γ, Perforin 1, and NKp46) via immunofluorescence imaging within the tumor sections. Overall, both abscopal tumors from resection and cryoablation had increased frequencies of CD8^+^ T cells compared to baseline resected tumors and an elevated IFN-γ signature, however these appeared to be equal between treatment groups (**Supplementary Figure 4A**). Interestingly, localization analysis indicates higher accumulation of CD4^+^ T cells and IFN-γ staining in the center of abscopal tumors from cryoablation, which suggests cryoablation induces better infiltration of abscopal tumors by select cell types (**Supplementary Figure 4B**). No clear differences were observed for Perforin 1 and NKp46 expression by immunofluorescence (**Supplementary Figure 5**). Representative images of each channel are presented in **Supplementary Figure 6**.

Lastly, we used Gene Set Enrichment Analysis (GSEA) to gain deeper insights on how cryoablation differentially modulates the abscopal immune response compared to resection. We found cryoablation led to enrichment of the inflammatory response, as well as the IL-2-STAT5 and IL-6-JAK-STAT3 signaling pathways at the abscopal tumors, compared to resection (**Figure 8E**). These findings combined with the flow cytometry results suggest that the increased inflammatory response occurring at the cryoablated tumors (**Figure 3**) might also lead to an enhanced inflammatory response at the abscopal tumors. Complimentary IPA analysis showed that amongst the top 20 immune pathways influenced by cryoablation and resection, there is downregulation of genes involved with TREM1, phagosome formation, and IL-8 signaling (**Supplementary Figure 7A**). Altogether, these data agree with previous studies showing the potential of cryoablation to generate the abscopal effect and show for the first time that as early as one-week after cryoablation, many anti-tumoral pathways are affected in distant (abscopal) tumors, in a mouse model of TNBC.

### 3.6 Deconvolution analysis of resected tumors by Cibersort can help guide therapeutic decisions

Cibersort is a computational method for estimating the immune composition of a sample from bulk tissue gene expression profiles, by combining support vector regression with prior knowledge of expression profiles from purified leukocyte subsets. We found that deconvolution analysis of bulk tissue gene expression, obtained from tumor sections prepared in slides, allows observation of the frequency of multiple immune populations (**Supplementary Figure 7B**). The ability to determine the frequency of immune cells for resected tumors using Cibersort demonstrates how this technique could be translated to the clinic with primary tumor biopsies to provide additional TIL information to help select potential candidates and guide treatment decisions.

## 4 Discussion

Surgically resecting aggressive tumors while maintaining cancer-free margins remains the mainstay of local therapy, however, the eventual prognosis largely dictated by tumor biology remains poor (43); though chemotherapeutic agents and radiation may aid in that scenario (44). Despite recent treatment advancements, particularly with the use of immunotherapeutic agents, TNBC patients remain at high risk for recurrence and metastasis (14–17) with addition of toxicities from these newer agents. Thus, strategies that elicit an improved anti-tumor immune response and overcome resistance are necessary for advancing the current standard of care. Cryoablation is a promising alternative to resection, since it kills the tumor cells and can provide a vast repertoire of preserved tumor cell associated antigens to boost the activation of immune cells (21). The activated anti-tumor T cells can then recognize both local and potential distant metastatic tumor cells. While cryoablation is already in clinical use to treat small/low-risk breast tumors, its clinical efficacy for TNBC remains unknown. Our prior study using animal models suggests cryoablation is a suitable substitute for resection, possessing the same ability to kill the tumor mass, while generating a superior immune response, characterized by increased infiltration of abscopal tumors by lymphocytes, and decreased metastasis rate at six weeks after the treatment (29). However, the early markers and the mechanisms that lead to the improved response are still unknown and knowledge of those will aid in the development of combinational therapies for cryoablation and pave the way for its use for TNBC.

Here, we show for the first time in a mouse model of TNBC that systemic changes are observable as early as one-week post-cryoablation. One such effect was reflected in tumor weights, with abscopal tumors from mice with cryoablated primary tumors presenting a significantly lower weight on day of sacrifice, than abscopal tumors from resection. The ability of cryoablation to induce superior control of tumor volume distantly at this early time-point suggests that the approach might result in improved surveillance for metastasis, which could significantly extend survival of TNBC patients, since currently only about 12% of patients with distant disease survive 5 years after diagnosis (18). Immune profiling by flow cytometry supports a model that early changes in immune cell frequencies contribute to the greater abscopal tumor elimination in mice from the cryoablation group. For example, we observed a significant increase in the frequency of NK cells in the abscopal tumors from cryoablation compared to abscopal tumors from resection, and trends for having more CD4^+^ and CD8^+^ effector/effector memory T cells.

NK cells are cytotoxic innate lymphocytes that can recognize and destroy tumor cells independently of major histocompatibility complex class I (MHC-I) molecules (45, 46). Tumor cells often downregulate the expression of MHC-I to avoid recognition by CD8^+^ T lymphocytes. Elegantly, NK cells activate when engaged with MHC-I-null cells resulting in direct tumor cell killing (46). Besides monitoring for the expression of MHC molecules, NK cells also detect stress-triggered self-ligands, which makes them able to recognize tumor cells even when they have retained MHC-I expression (47, 48). Therefore, having NK and T cells present in the tumor microenvironment is a powerful combination that allows the elimination of the tumor cells, where one cell can suffice this function when the other is not able to. Sabel et al. observed that NK cells obtained from the spleen of mice one-week after breast tumor cryoablation presented increased cytotoxic activity *in vitro* (30). In combination with our *in vivo* data, this suggests that cryoablation may influence the frequency and activity of NK cells. Future studies should evaluate the cytotoxic tumor killing ability of NK cells obtained from the abscopal tumors to completely elucidate this mechanism.

The molecules CD62L and CD44 have traditionally been used in flow cytometry to classify T cells into different subsets. Recently, a group defined the rarely studied double-negative (CD62L^-^, CD44^-^) CD4^+^ and CD8^+^ T cells as “pre-effector” cells, which have evidence to originate from naïve cells and can give rise to effector/effector memory T cells (33). In our results, we observed a significant decrease in the frequency of pre-effector CD4^+^ and CD8^+^ T cells in abscopal tumors from cryoablation, which was accompanied by a trend for having more effector/effector memory CD4^+^ and CD8^+^ T cells, compared to abscopal tumors from resection. These data suggest that cryoablation of the primary tumor might accelerate the development of effector/effector memory T cells in the abscopal tumors compared to resection.

The superior activation of CD4^+^ and CD8^+^ T cells could be induced by a higher frequency of migratory cDC1s (XCR1^+^ CD103^+^), which possess the unique ability to transport tumor antigens to lymphoid structures and excel at cross-presenting the TAAs to CD8^+^ T cells (49). At one-week post-cryoablation, we did not find statistical increases for those populations in the tumors. Nevertheless, we observed an overall trend for having more migratory DCs in the abscopal tumors and TdLNs in mice treated with cryoablation, and a significant increase in the frequency of that population in the spleens. Considering that one DC has the ability to scan over 500 T cells per hour (50), minor increases in their frequency may improve important T cell responses. Our results suggest the increased frequencies of migratory cDC1s may be one of the mechanisms resulting in heightened frequencies of effector/effector memory T cells. This model agrees with previous studies supporting the hypothesis that cryoablation improves anti-tumor immunity by recovering the function of DCs present in TdLNs (51) and enhancing the migration of DCs from the tumor to the TdLN (52). Further evidence that cDC1s play a role in the cryoablation-generated abscopal effect is supported by previous studies with other tumor models in which cryoablation in combination with dendritic cell therapy increased the anti-tumor immune response (53, 54). Indeed, the profile of immune cells in the axillary TdLNs is known to provide prognostic value for breast cancer patients, with increased CD4^+^ T cells and CD1a^+^ DCs correlating with higher rates of disease-free survival (55).

Another mechanism that may contribute to the improved anti-tumor response is the reduction of immunosuppressive signals in the abscopal tumors from cryoablation, partly provided by myeloid cells. We observed a significant decrease in the frequency of M1-like macrophages, and an overall trend for having fewer Ly6G^int^ neutrophils, immature macrophages, and inflammatory monocytes. M1-like macrophages are classically defined as anti-tumoral, while M2-like macrophages are pro-tumoral; however, these are only two states along a spectrum of different macrophage phenotypes (56, 57). For breast cancer, previous studies showed that having higher macrophage density (regardless of phenotype) correlated with poor prognosis (58, 59); therefore, having fewer M1-like macrophages at the abscopal tumors could be beneficial. Combined with the decreased immature macrophages and inflammatory monocytes signature, which are also considered pro-tumoral (60–63), the results suggest cryoablation alleviates immunosuppression at the abscopal tumors compared to those from resection. Importantly, Ly6G^int^ Siglec-F^hi^ neutrophils were established as immunosuppressive in lung cancer and acute spleen infections (34–36), and the trend for having decreased frequencies of those cells at the abscopal tumors from cryoablation corroborates the above-mentioned hypothesis, but further studies are needed to confirm the immunosuppressive activity of these cells in mouse breast cancer models. Additionally, it will be important to measure immunosuppressive activity of all populations mentioned here, to understand whether cryoablation is affecting functionality as well, or exclusively recruitment.

Besides the significant increase in the frequency of migratory cDC1s in the spleen of cryoablated mice, other immune cell frequencies were similar in abscopal tumors from cryoablation and resection in the spleen and blood. Our findings agree with results from Sabel et al., which showed that tumor-specific T cell responses after cryoablation were present locally (TdLN) with no major changes in the periphery (spleen), with the caveat that this study examined only cytokine secretion and cytotoxic activity, and not immune cell frequencies (30). Interestingly, we observed a trend for reduced systemic central memory CD4^+^ T cells. Central memory T cells are important to provide a fast and augmented immune response upon secondary exposure to previously encountered antigens and, therefore, are desirable in the tumor context. However, since our analysis was carried out to evaluate early events, it was expected that there would not be increased frequencies of central memory CD4^+^ T cell generation with cryoablation and it would be more appropriate to analyze this population at later time points in future studies.

We used RNA-seq analysis to identify potential mechanisms of the early immune response leading to an enhanced abscopal effect induced by cryoablation compared to resection. Results confirm that it is possible to observe the abscopal effects of cryoablation as early as one-week after the procedure, with multiple pathways being affected by the treatment, such as leukocyte chemotaxis, lymphocyte activation and NK cell activation, which aligns with the flow cytometry data. Some of the upregulated genes at abscopal tumors from cryoablation could be underlying the differences found at the protein level by flow cytometry; the increased expression of *Il12b* and *Il2ra* might be one of the mechanisms leading to higher NK cell frequencies at the abscopal tumors by inducing their generation, recruitment, and activation (64, 65). The increased expression of *Pfr1* at abscopal tumors from cryoablation further suggests that NK/NKT and T cells at the TIME are potentially secreting more perforin, which could be one of the means leading to the decreased abscopal tumor weight, but additional functional experiments are needed to confirm this hypothesis. We observed that some of the DEGs in the abscopal tumors from cryoablation compared to those of resection suggest negative modulation of the anti-tumor activity, however, considering the decreased abscopal tumor size, increased infiltration by anti-tumor immune cells and increased anti-tumor gene signature, we believe that this could be part of a mechanism to prevent an exacerbated response that might cause damage to non-cancerous tissue.

Remarkably, GSEA analysis revealed upregulation of the IL-2/STAT5 and IL-6/STAT3 pathways in abscopal tumors from cryoablation compared to abscopal tumors from resection. Various studies showed that expression of STAT5 by tumor cells could act as either a tumor suppressor or oncogene in breast cancer under different circumstances (66), while STAT3 expression in tumor cells has mainly been associated with a pro-tumoral microenvironment (67). As discussed by Halim et al. (66), STAT5 signaling is thought to have oncogenic roles during tumor development, and tumor suppressor functions during tumor progression. Moreover, evidence suggests STAT5 has dominance over STAT3 (68) and that tumor suppressor activities of STAT5 can counteract the oncologic effects of STAT3 (69). Therefore, the results suggest that while also inducing higher STAT3 signaling, cryoablation induces STAT5 signaling, which has the potential to inhibit STAT3 oncogenic signaling and lead to reduced tumor cell proliferation, higher sensitivity to chemotherapy (69), and decreased angiogenesis, tumor cell migration, and metastasis (70, 71). This could be very important for breast cancer, since STAT3 is known to promote breast cancer malignancy and clinical trials have yet to show successful inhibition of its pro-tumoral effects; in that sense, cryoablation could make TNBC more amenable to STAT3-inhibitor treatment, however, confirmational analyses are needed to support these findings.

Under current standard of care practices, it is near impossible to do routine flow cytometry analysis for tumor immune cell infiltrate characterization and so H&E TIL counts are currently used for that purpose with biopsies (72). However, H&E TIL counts does not allow analysis of lymphocyte subpopulations. To identify immune populations without flow cytometry, we used an approach that would be technically achievable based on clinical protocols. We used tissue sections from histology slides (representative of tumor biopsy) for the RNA-seq analysis and deconvolution of the immune cells using Cibersort analysis, representing the most pragmatic clinical approach. We found that this technique allowed identification of multiple and different immune populations for each individual mouse. The goal of this approach was to develop a theoretical approach that can provide insight into the TIME that could be translated to clinical application for identifying best-fit TNBC candidates for cryoablation and to develop appropriate combinational therapies, such as use of immune checkpoint inhibition (ICI).

Lumpectomy with surgical axillary followed by adjuvant treatments is recommended for early-stage breast cancer by the National Comprehensive Cancer Network (NCCN) 2020 guidelines (73). Cryoablation is currently in use for small, low-risk tumors for patients who are not candidates for standard treatment or woman who refuse surgery, and is advantageous over other ablative techniques, such as laser thermotherapy and radiofrequency ablation (RFA), since it causes minimal discomfort and can be performed in the office with local anesthesia, while the others have to be performed in the radiology department, and require anesthesiology-administered sedation for pain control (74). ICE3, a recently completed prospective, multicenter trial found that hormone receptor (HR)-positive and human epidermal growth factor receptor 2 (HER2)-negative invasive ductal carcinomas (IDC) treated with cryoablation had a decreased 5-year overall recurrence compared to surgical resection. Subset analyses on patients additionally treated with adjuvant endocrine therapy and combination adjuvant endocrine and radiation therapy found decreased recurrence (75). There are clinical trials investigating cryoablation combined with ICI therapy, as well. With such a promising response in less immunogenic breast cancer, cryoablation could be the key to improving patient outcomes with TNBC. As of August 2024, there were 3 active trials investigating cryoablation in combination with immunotherapy in TNBC on ClinicalTrials.gov, 2 recruiting and 1 not yet recruiting, all utilizing pembrolizumab and one additionally utilizing ipilimumab and nivolumab. Patient selection is rooted in TNBC status and while these results will be interesting to see, screening patients further utilizing molecular profiling could help predict which patients may best benefit from cryoablation and checkpoint inhibition combination therapy, since triple negative tumors encompass both basal-like and non-basal cancers on genomic profiling (76). Further investigation into this would allow individualized treatment with the greatest impact in a poor-prognosis disease.

Although promising results were observed in the animal experiments reported here, some caveats are worth highlighting. Since mice are small, the cryoablation technique had to be adapted. In humans, the procedure is minimally invasive, where the probe is inserted into the breast tumor through a small incision in the skin with guidance from imaging techniques, such as ultrasound; a saline hydro dissection procedure is used to prevent skin frostbite (77). Meanwhile, in mice it is necessary to surgically retract the skin to expose the tumor and, instead of trying to insert the probe into these small tumors, it is laid directly on top of the tumors for freezing (**Figure 2A**). We acknowledge these modifications make it possible for the surgery to play a role in our results; however, based on the resection controls, we still see significant changes in the immune response to cryoablation compared to resection. Wu et al. showed similar changes in the abscopal tumors as observed in our study, using an analogous cryoablation technique for a model of colon cancer (31).

Besides its application for breast cancer, cryoablation has also been used to treat benign and malignant liver, colon, prostate, skin, kidney, lung and bone tumors, as well as non-tumor heart diseases (78, 79), with advantages and shortcomings involved. Similarly to results observed for breast cancer, cryoablation was shown to be just as effective as resection for small tumors (22, 79–82); however, for larger or multifocal tumors, cryoablation showed decreased efficiency, with higher rates of tumor progression and adverse events (83–86). Therefore, it seems that combination of cryoablation with other therapies would also be ideal for other tumor types besides TNBC, however, additional research is needed to elucidate the immune effects of the technique in those different microenvironments.

Overall, we observed an improved anti-tumor response due to primary tumor cryoablation. Our gene expression results were reflective of those changes and may provide physicians with clinically actionable information, such as knowledge of genes and pathways affected by cryoablation, which could be implemented to guide decisions for best therapy strategies. In that scenario, a promising follow-up therapy is the combination with immune checkpoint inhibitors, since those will allow sustained activation of effector cells (21, 87). The greater availability of tumor antigens and boosted inflammatory signals offered by cryoablation, combined with the relief of immunosuppressive signals provided by the immune checkpoint inhibitors, may result in a strong enough response to safely treat high-risk breast cancer.

## 5 Conclusion

Cryoablation induces a systemic abscopal effect, leading to increased frequencies of cytotoxic immune cells at distant tumor sites, which is accompanied by higher frequencies of migratory dendritic cells in the spleen, detectable as early as one-week after the procedure. Gene expression analysis supports those findings and reveals genes and pathways affected by cryoablation, providing the field with new information that could assist in developing combinational therapeutic approaches with cryoablation and potentially help guide therapy decisions.

## Data Availability

The data discussed in this publication have been deposited in NCBI's Gene Expression Omnibus (88) and are accessible through GEO Series accession number GSE282249 (https://www.ncbi.nlm.nih.gov/geo/query/acc.cgi?acc=GSE282249).
