# Supplementary material for: Cryoablation of primary breast cancer tumors induces a systemic abscopal effect altering TIME (Tumor Immune Microenvironment) in distant tumors
Source: Front Immunol. 2024 Dec 2;15:1498942. doi: 10.3389/fimmu.2024.1498942 (PMC11657241; doi:10.3389/fimmu.2024.1498942)
Supplement: Supplementary file 1 [file DataSheet1.docx]

Supplementary Material

# Supplementary methods

**Immunofluorescence for immuno‑profiling and cellular spatial analysis.**

Hits from our RNA-seq analyses were analyzed by quantitating tumors for T cell markers by staining with immunofluorescent antibodies against CD4, CD8, INFγ, perforin, and NKp46 (Supplementary Table 1). Tumor sections were deparaffinized, underwent heat-induced antigen retrieval, blocking for 1.5 hours at room temperature (RT) with blocking buffer (PBS, 0.1% Saponin, 5% BSA), and were incubated overnight at 4⁰C in blocking buffer with the primary antibodies. The next morning, slides were washed with PBS and incubated 2 hours at RT with an Alexa Fluor 647-conjugated secondary donkey anti-goat IgG (A-21447; Invitrogen) at 1:200, washed and then incubated for another 2 hours at RT with an Alexa Fluor 488-conjugated secondary goat anti-rat IgG (A-11006; Invitrogen) at 1:200 dilution, and an Alexa Fluor 568-conjugated secondary goat anti-rabbit IgG (A-11036; Invitrogen) at 1:2000 dilution. Then, slides were washed and incubated with Vector TrueView (SP-8500, Vector Laboratories) to quench autofluorescence and mounted with VECTASHIELD Vibrance Antifade Mounting Medium with DAPI (H-1800, Vector Laboratories). Samples were imaged using a confocal microscope Nikon T-1E with a 20x objective and NIS software, then analyzed using Fiji:ImageJ 1.54f software [1]. Antibody fluorescence was normalized by DAPI staining for each sample.

# Supplementary Tables and Figures

**Supplementary Table 1. Antibodies used for immunofluorescence.**

|  | **Antibody** | **Host species** | **Clone** | **Catalog number** | **Manufacturer** | **Concentration** |
| --- | --- | --- | --- | --- | --- | --- |
| **Panel 1** | CD8 alpha | Rabbit | D4W2Z | 98941 | Cell Signaling | 1:50 |
|  | CD4 | Rat | 4SM95 | 14-9766-82 | Invitrogen | 1:50 |
|  | IFN gamma | Goat | Polyclonal | AF-585-SP | R&D Systems | 1:20 |
| **Panel 2** | CD8 alpha | Rabbit | D4W2Z | 98941 | Cell Signaling | 1:50 |
|  | Perforin | Rat | CB5.4 | ab16074 | Abcam | 1:100 |
|  | NKp46 | Goat | Polyclonal | AF2225 | R&D Systems | 1:100 |

**Supplementary Table 2. Differentially expressed genes.** Submitted in an excel file.

**
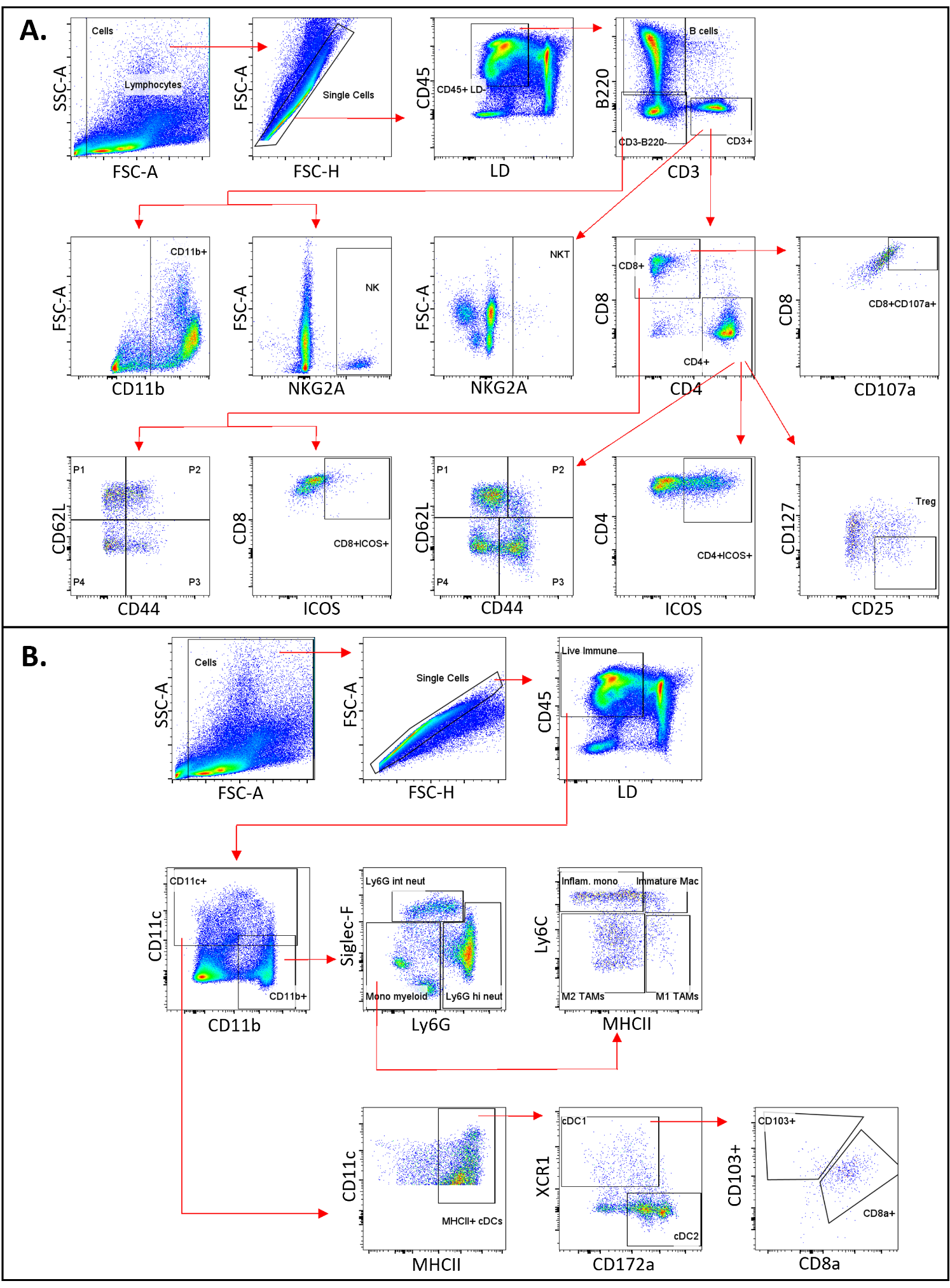
**

**SF1. Flow cytometry gating strategy.** Debris and doublets were excluded, and live immune cells (CD45^+^ LD^-^) were selected in the beginning of all analysis. A) Gate strategy for the lymphocyte panel. B cells were defined as B220^+^, myeloid cells were CD11b^+^, NK cells were NKG2A^+^, and T cells were CD3^+^. On the CD3^+^ population, we further gated on NKG2A^+^ (NKT cells), CD4^+^ (helper T cells), and CD8^+^ (cytotoxic T cells) events. CD4^+^ and CD8^+^ T cells were considered activated based upon ICOS expression, and subpopulations were defined based on the expression of CD62L and CD44, where: CD62L^+^CD44^-^ are Naïve T cells (P1); CD62L^+^CD44^+^ are central memory T cells (P2); CD62L^-^CD44^+^ are effector/effector memory T cells (P3); and CD62L^-^CD44^-^ are pre-effector T cells (P4). Regulatory T cells (Treg) were defined as CD4^+^, CD127^low^, and CD25^high^, and CD8^+^ T cells who secreted perforin were CD107a^+^. B) Gate strategy for the myeloid panel. Conventional dendritic cells (cDC) were defined as CD11c^+^ and MHCII^high^ and then assessed to separate XCR1^+^ type 1 and CD172α^+^ type 2 conventional dendritic cells. Type 1 cDC were further classified into migratory and resident cells based on CD103 and CD8α expression, respectively. The CD11b^+^ Ly6G^+^ population was defined into 2 subpopulations: Ly6G^high^ (Siglec-F^low^) and Ly6G^intermediate^ (Siglec-F^high^) neutrophils. Finally, CD11b^+^, Ly6G^-^, Siglec-F^-^ monocytes and macrophages were defined based on Ly6C and MHCII expression: M2-like TAMs were defined as Ly6C^low^ and MHCII^low^; M1-like TAMs were Ly6C^low^ and MHCII^high^; inflammatory monocytes were Ly6C^high^ and MHCII^low^; and immature macrophages were Ly6C+ and MHCII^high^.

**
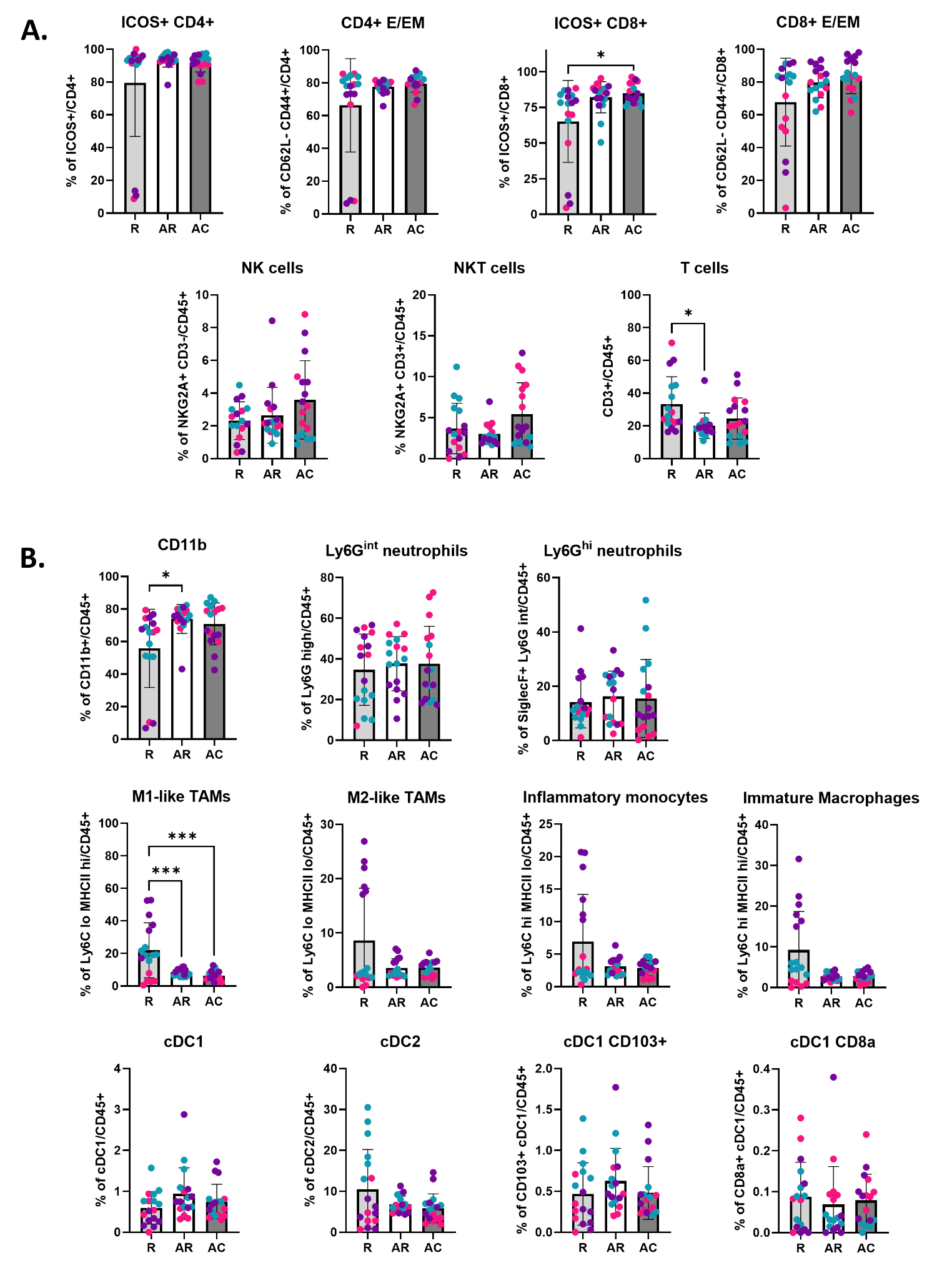
**

**SF2. Raw flow cytometry data for abscopal and resected tumors.** A) Lymphoid populations at the primary tumors. Subpopulations of T cells were analyzed as frequency of the parent (CD4^+^ or CD8^+^), while the parent immune populations were analyzed as frequency of the live immune cells (CD45^+^). B) Myeloid populations at the primary tumors, analyzed as frequency of the live immune cells (CD45^+^). The different dot colors indicate the experiment. One-way ANOVA for normally distributed data or Kruskal Wallis test for non-normally distributed data was performed comparing resected and abscopal tumors, with p<0.05 (*) considered significant. R = resected tumors; AR = abscopal tumors from resection; AC = abscopal tumors from cryoablation. N = 17.


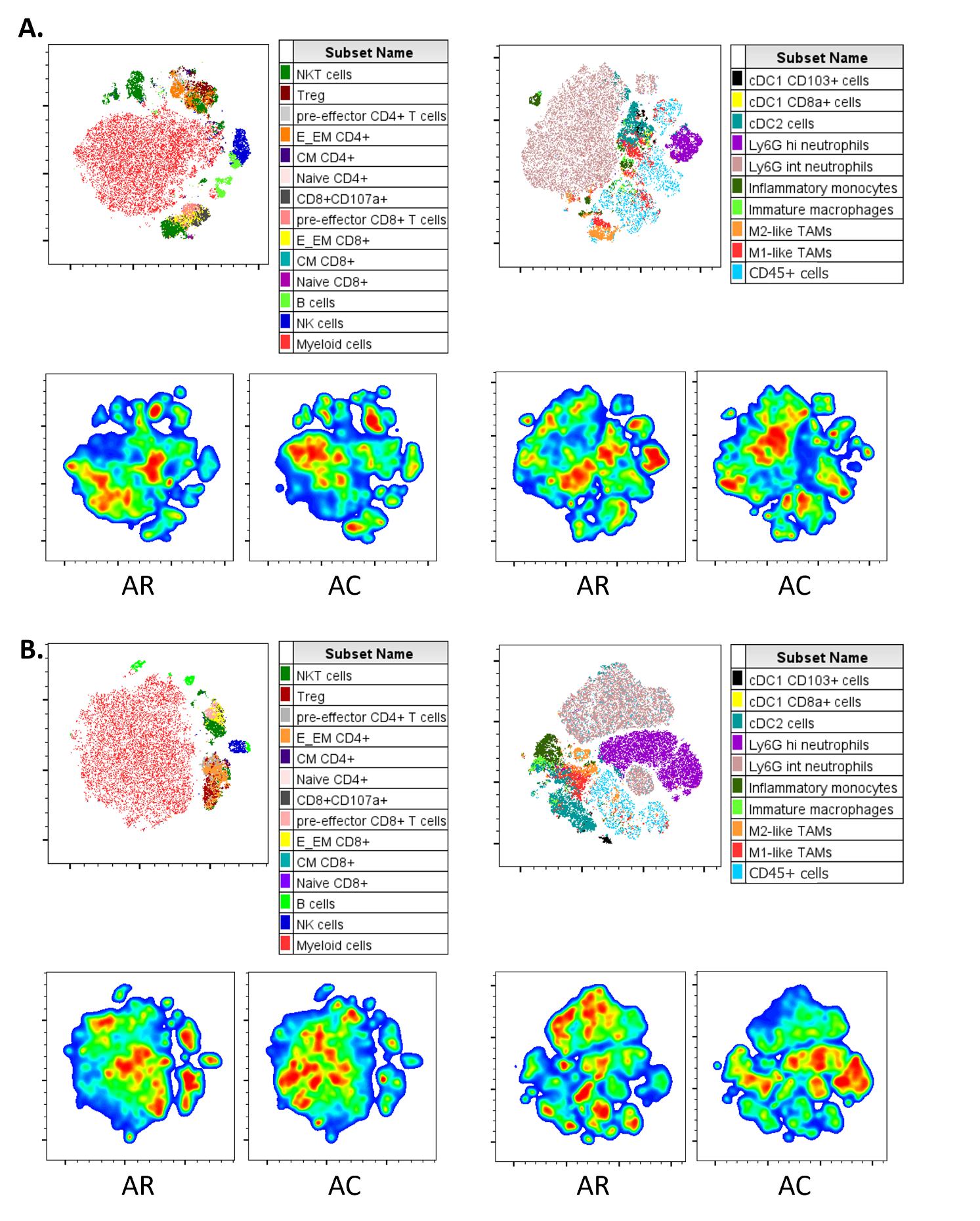


**SF3. tSNE maps from other experimental replicates.** Figure shows tSNE maps and heatmaps from other two experimental replicates (A and B), for the lymphoid (left) and myeloid (right) panels. AR = abscopal from resection; AC = abscopal from cryoablation; CM = central memory; E/EM = effector/effector memory. n per experiment = 5-6.


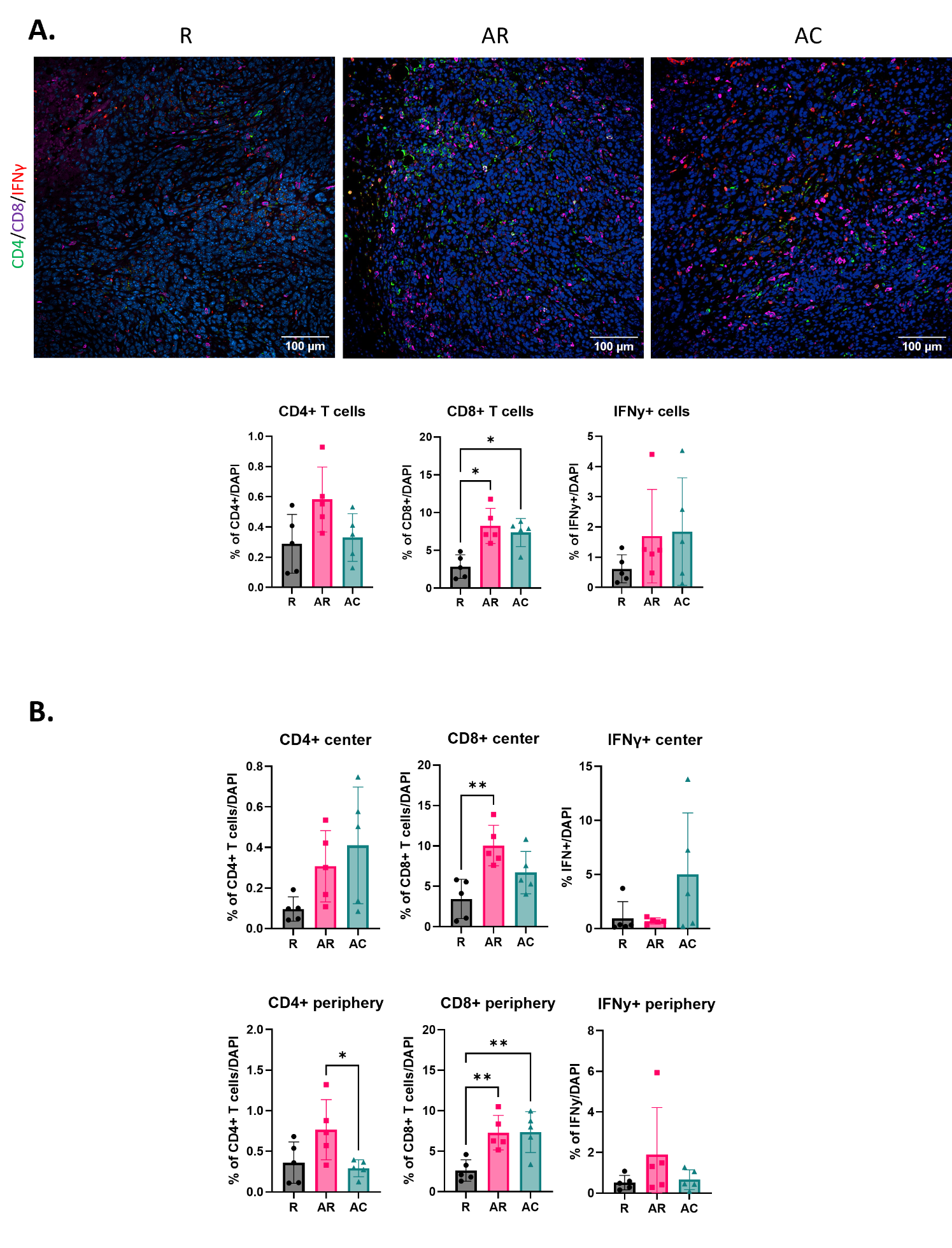


**SF4. CD4, CD8, and IFNγ immunofluorescence.** Resected and abscopal tumor paraffinized sections were stained with anti-CD4, CD8 and IFNγ for immunofluorescence. A) Top: merged images; Bottom: quantification of average staining. B) Quantification based on location; top: center; bottom: periphery. All images were taken at 20X magnification. One-way ANOVA for normally distributed data or Kruskal Wallis test for non-normally distributed data was performed comparing resected and abscopal tumors, with p<0.05 (*) AND P<0.01 (**) considered significant. R = resected tumors; AR = abscopal tumors from resection; AC = abscopal tumors from cryoablation. n = 5.


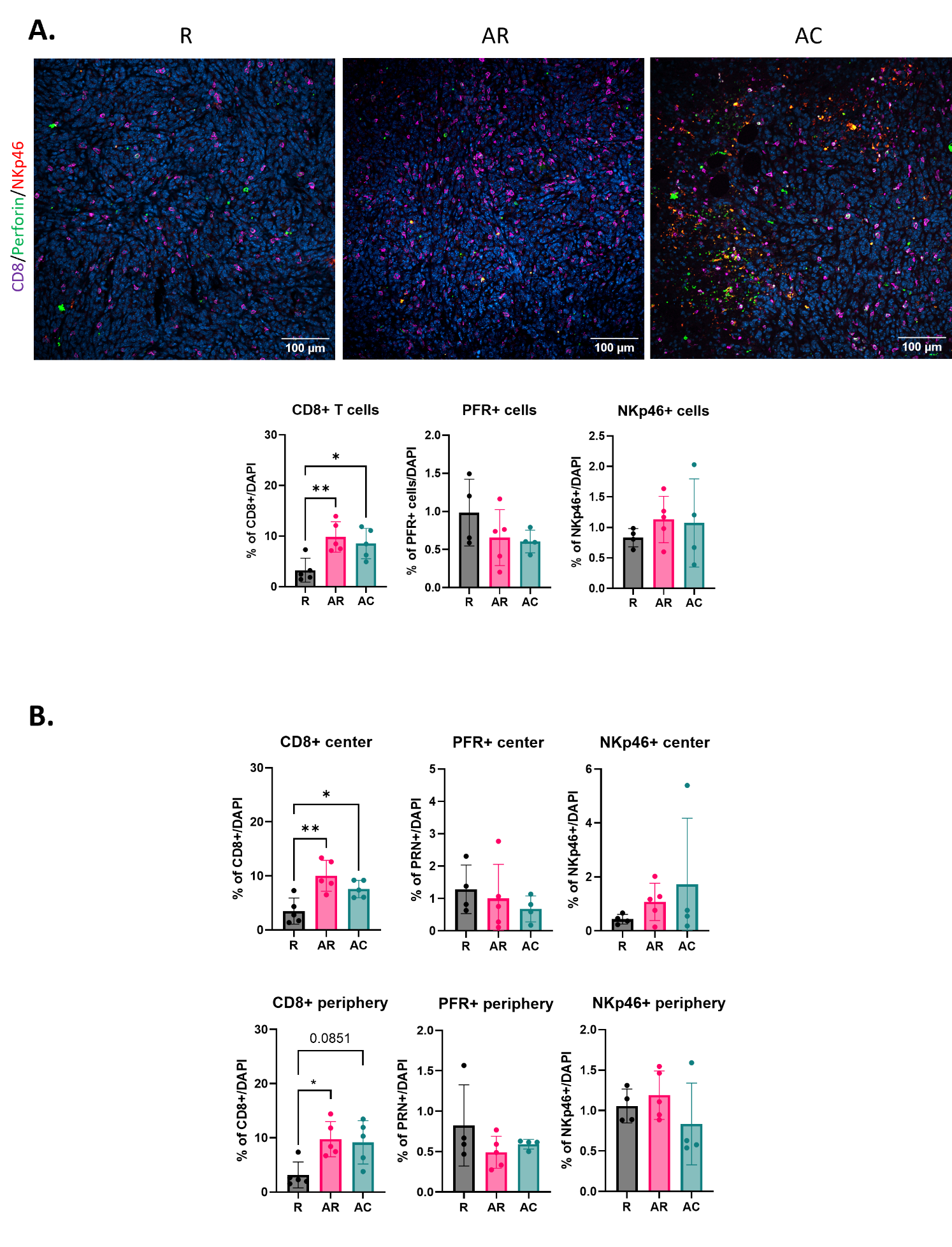


**SF5. CD8, PFR, and NKp46 immunofluorescence.** Resected and abscopal tumor paraffinized sections were stained with anti-CD8, Pfr and NKp46 for immunofluorescence. A) Top: merged images; Bottom: quantification of average staining. B) Quantification based on location; top: center; bottom: periphery. All images were taken at 20X magnification. One-way ANOVA for normally distributed data or Kruskal Wallis test for non-normally distributed data was performed comparing resected and abscopal tumors, with p<0.05 (*) AND P<0.01 (**) considered significant. R = resected tumors; AR = abscopal tumors from resection; AC = abscopal tumors from cryoablation. n = 4-5.


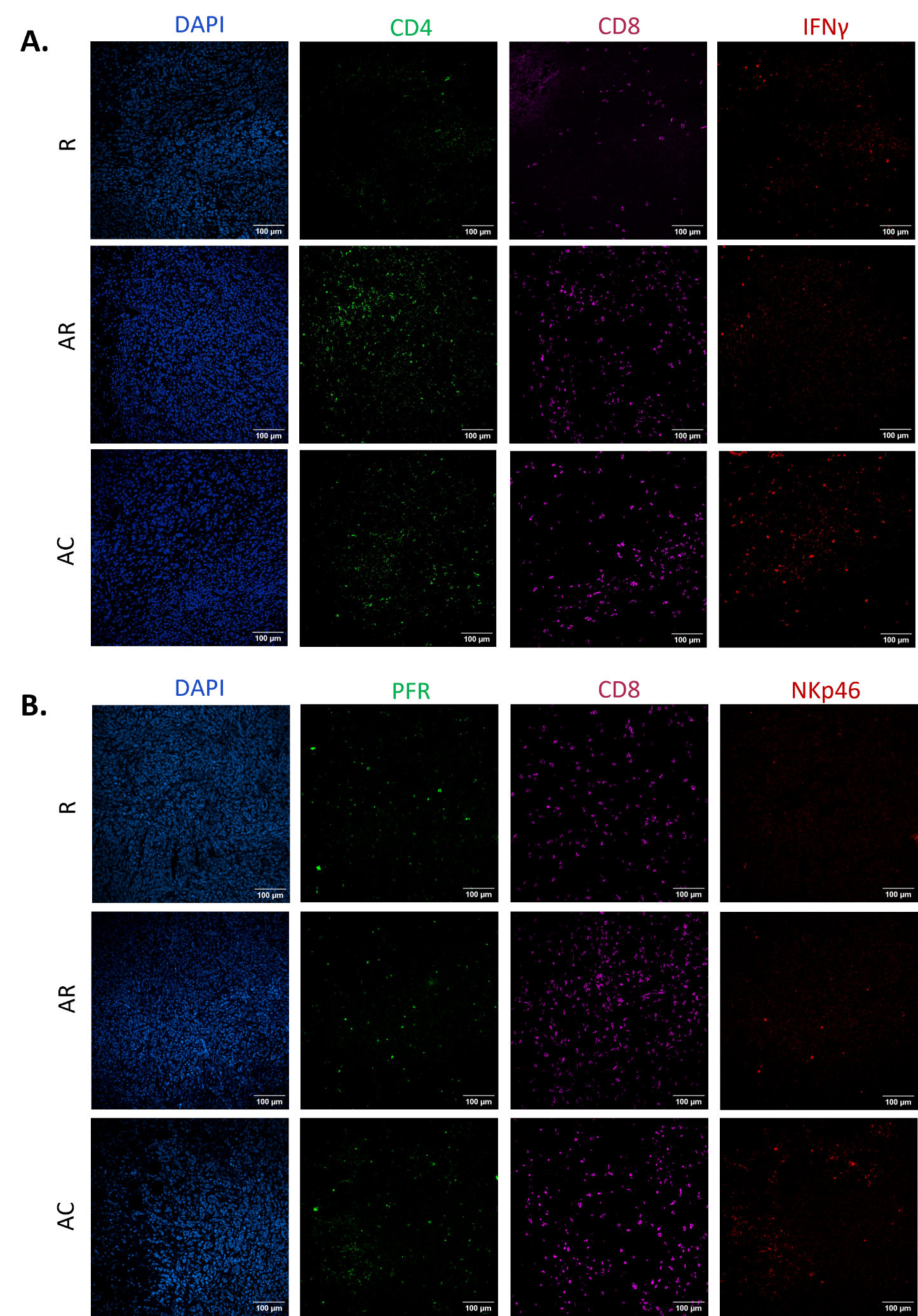


**SF6. Individual images for each channel from immunofluorescence analysis.** A) From left to right: DAPI (blue), CD4 (green), CD8 (magenta), and IFNγ (red). B) From left to right: DAPI (blue), Pfr (green), CD8 (magenta), and NKp46 (red). All images were taken at 20X magnification. R = resected tumors; AR = abscopal tumors from resection; AC = abscopal tumors from cryoablation. n = 4-5.


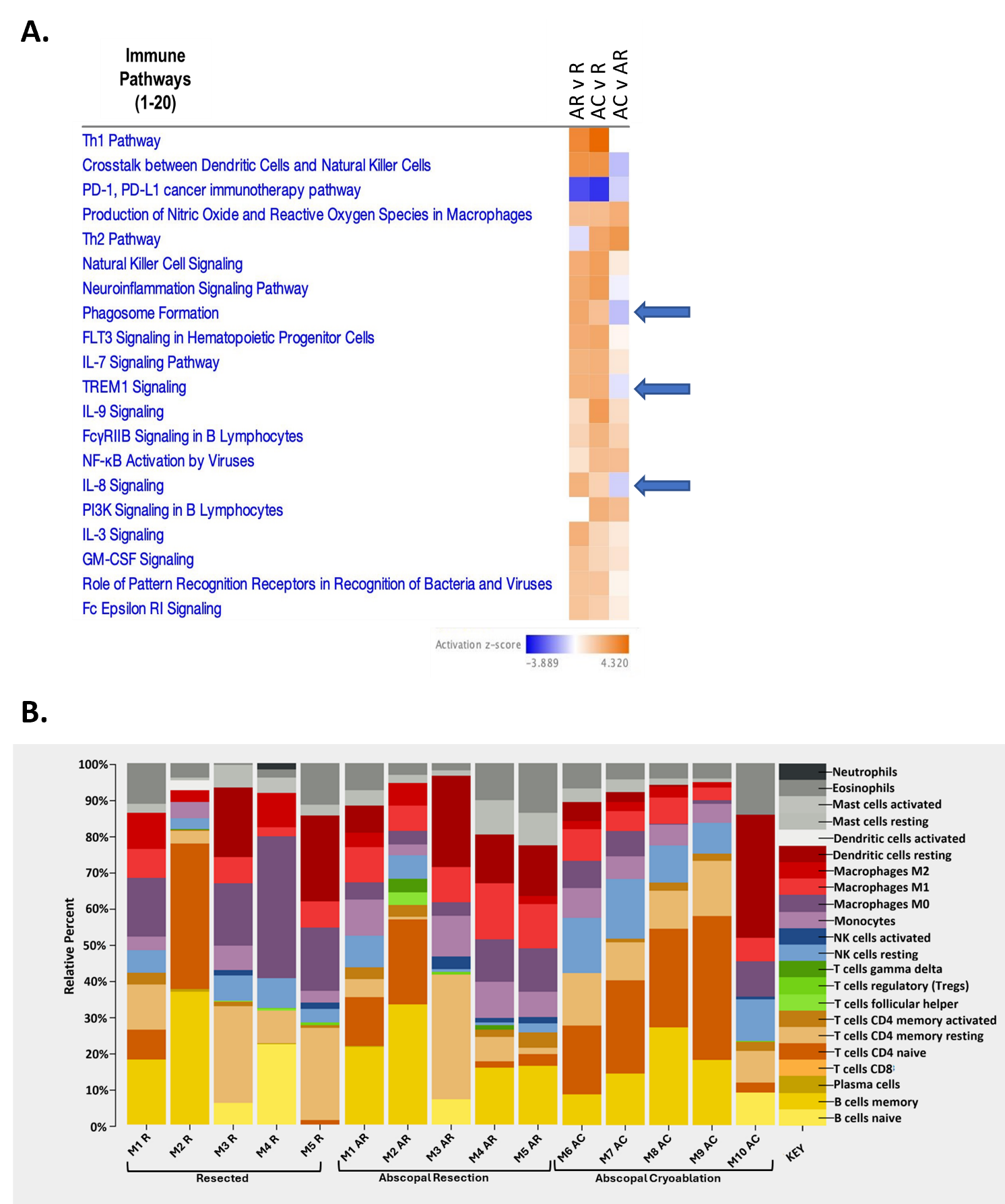


**SF7. Differentially expressed immune function-related signaling pathways and genes and deconvolution analysis by Cibersort.** **(A)** To identify potential immune-related pathways, comparison analyses were performed specifically on cellular immune response, cytokine signaling, and humoral immune response pathways using IPA. The top 20 differentially activated “immune” pathways are shown in the heatmap. **(B)** Deconvolution of data was performed using Cibersort to estimate the immune composition of bulk tumors. Immune populations are shown in different colors. The X axis shows the individual mice, while the Y axis shows the relative percent of each immune population. R = resected tumors; AR = abscopal tumors from resection; AC = abscopal tumors from cryoablation. n = 5.

**References**

1. Schindelin, J., et al., *Fiji: an open-source platform for biological-image analysis.* Nat Methods, 2012. **9**(7): p. 676-82.
